# Electrical stimulation for brighter persistent luminescence

**DOI:** 10.1038/s41377-024-01507-0

**Published:** 2024-07-15

**Authors:** Xilin Ma, Yuhua Wang, Takatoshi Seto

**Affiliations:** Key Laboratory for Special Function Materials and Structural Design of the Ministry of Education, National & Local Joint Engineering Laboratory for Optical Conversion Materials and Technology of National Development and Reform Commission, Department of Materials Science, School of Materials and Energy, Lanzhou University, No. 222, South Tianshui Road, Lanzhou, Gansu 730000 China

**Keywords:** Photonic devices, Inorganic LEDs

## Abstract

An immature understanding of the mechanisms of persistent luminescence (PersL) has hindered the development of new persistent luminescent materials (PersLMs) with increased brightness. In this regard, in-situ direct current (DC) electric field measurements were conducted on a layered structure composed of the SrAl_2_O_4_:Eu^2+^,Dy^3+^ phosphor, and an electrode. In this study, the photoluminescence (PL) and afterglow properties were investigated with respect to voltage by analyzing the current signal and thermoluminescence (TL) spectroscopy. The intensity of PersL increased due to a novel phenomenon known as “external electric field stimulated enhancement of initial brightness of afterglow”. This dynamic process was illustrated via the use of a rate equation approach, where the electrons trapped by the ultra-shallow trap at 0.022 eV could be transferred through the conduction band during long afterglow. The afterglow intensity could reach 0.538 cd m^−2^ at a 6 V electric voltage. The design of an electric field stimulation technique enables the enhancement of the intensity of PersLMs and provides a new perspective for exploring the fundamental mechanics of certain established PersLMs.

## Abstract (web summary)

An electric field is applied to a layered device comprising the persistent phosphor. A novel phenomenon, namely the electrical stimulation for brighter persistent luminescence, is demonstrated.

## Graphical abstract

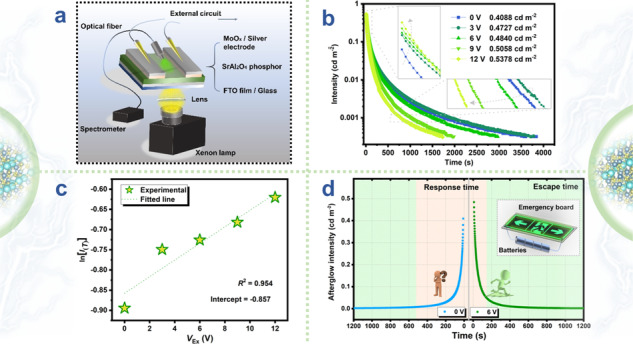

## Introduction

Persistent luminescence (PersL) is called “afterglow” or “long-lasting luminescence” and is characterized by an extremely long decay lifetime and has been widely utilized in plates guiding refuge at night emergencies, clock displays in dark rooms, biomarkers in the medical field, etc^1,2^. Since Matsuzawa et al.^3^ first successfully developed a new yellow-green-emitting PersLM, SrAl_2_O_4_:Eu^2+^,Dy^3+^ (abbreviated as SAO:Eu^2+^,Dy^3+^), in 1996, the study of novel PersLMs began to accelerate^4–7^. Most importantly, a wide variety of lanthanide ion-doped PersLMs have been explored: CaAl_2_O_4_:Eu^2+^,Nd^3+^ (blue)^8^, Sr_2_MgSi_2_O_7_:Eu^2+^,Dy^3+^ (blue)^9^, Sr_4_Al_14_O_25_:Eu^2+^,Dy^3+^ (cyan)^10^, Ca_2_BO_3_Cl:Eu^2+^,Dy^3+^ (yellow)^11^, Sr_3_SiO_5_:Eu^2+^,Nb^5+^ (yellow)^12^, Y_2_O_2_S:Eu^3+^,Mg^2+^,Ti^4+^ (red)^13^, Sr_2_Si_5_N_8_:Eu^2+^,Tm^3+^ (red)^14^. Some of these PersLMs are effectively commercialized. However, according to Fig. S1, SAO:Eu^2+^,Dy^3+^ has outstanding performance and is still the most encountered. No other PersLMs powerful than SAO:Eu^2+^,Dy^3+^ have been developed; thus, the increased industrial demands for a “much brighter afterglow” have not been successfully met. When sudden power outages in buildings such as factories, commercial facilities, or business rooms occur, people need a much brighter and clearer indicator that can guide them quickly and safely to the exit or the shelter in the dark. Thus, a bright afterglow in a short time of ~20 min is needed rather than a relatively long time, such as 1–10 h^15–17^.

To obtain the PersLMs with higher brightness, it is necessary to start from the basic afterglow mechanism. Understanding the afterglow mechanism for Eu^2+^(Ce^3+^)-Ln^3+^ doped persistent phosphors, as represented in SAO:Eu^2+^,Dy^3+^, has shown gradual progress. The Matsuzawa model (carrier: hole, carrier path: valence band)^3,18^, Aitasalo model (carrier: hole)^19^, Dorenbos-Nakazawa (D-N) model (carrier: electron, carrier path: conduction band)^20,21^, and Clabau model (carrier: electron, carrier path: inside band gap)^22^ have been proposed. Among them, the D-N model has been considered as a possible mechanism, since this method has generally not contradicted the experimental results. Ueda et al. observed unique photocurrent peaks similar to the two main excitation peaks corresponding to the 4f → 5d transition at ~350 nm and 425 nm in the Ce^3+^-activated Ga-substituted garnet-based phosphors, and the intensity of the photocurrent peaks became stronger with increasing substituted Ga causing stronger afterglow^23^. Moreover, Korthout observed a small change from Eu^2+^ to Eu^3+^ during the excitation process just before afterglow^24^. These results confirm half of the D-N model, where an excited electron moves between the Eu^2+^ 5d state and the trap center through the conduction band during the excitation process and the afterglow process. In the other half of the D-N model, the above electron trap center is not a defect or vacancy caused by the substitution of Ln^3+^; rather, Ln^3+^ receives an electron and, becomes Ln^2+^ in the excitation process, and then Ln^2+^ releases an electron to Eu in the afterglow process. The detection of Ln^2+^ during the afterglow process has been achieved; for example, Joos et al. conducted the photo-stimulated compensation and detected a small amount of Dy^2+^ for the excitation in Sr_4_Al_14_O_25_: Eu^2+^, Dy^3+^ during an X-ray scan^25^. However, understanding the behavior of PersL requires additional experimental data and new methods, particularly to clarify how the carrier transfer paths can enhance the intensities of the afterglow^26–28^.

In 2023, we (Peng et al.) observed the formation of Sm^2+^ in BaZrSi_3_O_9_: Eu^2+^, Sm^3+^ after excitation under UV light to drive the afterglow^29^. Thus, the rate-determining step is the release of electrons from the trap center to the conduction band in the three steps of the electron cycle during the afterglow process:

1. Trap center to the conduction band.
2. Conduction band to 5d band of Eu^2+^.
3. 5d band of Eu^2+^ to 4f band of Eu^2+^ (emission).

The time for step (3) is quite rapid, at the microsecond level (decay time of 4f-5d transition of Eu^2+^), and step (2) is also quite rapid for the observation of both of the changes, Sm^3+^→Sm^2+^ and Eu^3+^→Eu^2+^; these changes perfectly coincide with each other at time-scale of more than 10 s without any time delay in the change in Eu^3+^→Eu^2+^ for the change in Sm^3+^→Sm^2+^. The time delay corresponds to the time needed for the electron to move from the Sm part to the Eu part through the conduction band. Since the above mechanism of afterglow has a long history, we consider that a key factor for strong afterglow emission is the process in which electrons are transferred from the trap center to the conduction band and that free electrons are responsible for at least a part of this process.

Herein, we attempted to obtain a breakthrough for “further stronger afterglow” and began to apply an external electric field to the strongest persistent phosphor, SAO:Eu^2+^,Dy^3+^, currently known. An external electric field can produce an electron Coulomb force, which should significantly drive nearly free electrons to escape the energy level of the traps. By constructing phosphor/electrode layered structures, the properties of afterglow are monitored under the lower direct current (DC) electric field. The charge carrier behaviors and defect evolution are evaluated by electrical signals and spectroscopy techniques. This extraordinary strategy reveals a new perspective on long afterglow mechanisms and underlying physics, which can aid in the understanding of the key role of co-doped lanthanide ions and acquiring afterglow materials.

## Results

### Crystal structure and PL identifications

**Fig. 1:**
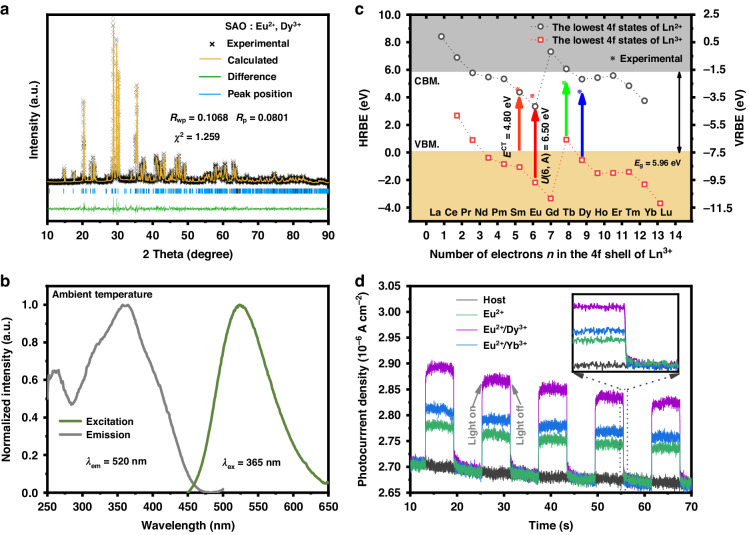
Crystal structure and PL identifications of SAO:Eu^2+^, Dy^3+^. **a** Rietveld refinement results of SAO:Eu^2+^,Dy^3+^. **b** The PLE and PL spectra of SAO:Eu^2+^,Dy^3+^. **c** The HRBE and VRBE diagram of the SAO sample. **d** The photocurrent signal of SAO host, single-doped SAO: Eu^2+^ and co-doped SAO:Eu^2+^,RE^3+^ (RE^3+^ = Dy^3+^, Yb^3+^) activated at 365 nm. Inset is the local zoomed image

Figure 1a shows that the series SAO:Eu^2+^,RE^3+^ used in this study had good single-phases (Rietveld Refinement results are listed in Table S1 and S2). The emission band in Fig. 1b was ascribed to the 4f^6^5d^1^ → 4f^7^ transition of Eu^2+^. This confirmed the introduction of Eu^2+^ in the SAO host, which was consistent with the results of other researchers^22,30^. The Host Referred Binding Energy diagram and Vacuum Referred Binding Energy (VRBE) diagram were subsequently constructed based on Fig. S3 and Dorenbos theory^31–35^, as exhibited in Fig. 1c; here, the 4f state levels of lanthanide ions were located nearly below the conduction band minimum (CBM), which could be treated as shallow donors (Pr^3+^, Dy^3+^, Ho^3+^, etc.) and deep donors (Sm^3+^, Tm^3+^, Yb^3+^, etc.)^36^. This result was consistent with the PersL decay curves and TL results shown in Fig. S4, Fig. S5, Tables S3, and Fig. S4. In Fig. 1d, the host SAO did not produce an evident photocurrent signal during an on-off cycle, but after Eu^2+^, Eu^2+^/Dy^3+^, and Eu^2+^/Yb^3+^ doping, the photocurrent signals could be detected, and the average value of Eu^2+^/Dy^3+^ was 2.8 times greater than that of SAO: Eu^2+^. This indicated that SAO:Eu^2+^,Dy^3+^ has appropriate trap depth and sufficient trap concentration, the carriers trapped in the shallow traps of Eu^2+^/Dy^3+^ near the CBM were the easiest to release into the conduction band, while deep traps were not.

### PL, PersL, and TL properties under electrical stimulation

The respective roles of the emission centers and trap centers are difficult to distinguish during the PersL process from the photocurrent signal in Fig. 1d, Fig. S6, and the steady PL spectra in Fig. S7. Accordingly, a low external DC electric field technique is proposed to investigate long afterglow and this technique relies on three main criteria:

1. There are a large number of traps at different depths caused by defects in the host materials^37^, and these traps can often become recombination center and affect the luminescent behavior (spectral shape, decay lifetime, et al.).
2. Detecting under a low DC electric field can help avoid ionization of the SAO:Eu^2+^,RE^3+^ phosphor and improve the estimation of whether the nature of the carriers in the afterglow is directly transferred between lanthanide ions or through the conduction band.
3. The existence of the traps often influences the carrier migration behavior inside the materials (conductivity, photoconductivity, etc.), and the dynamic properties of traps can be comprehensively inferred by combining current singles with other conventional means^38,39^.

**Fig. 2:**
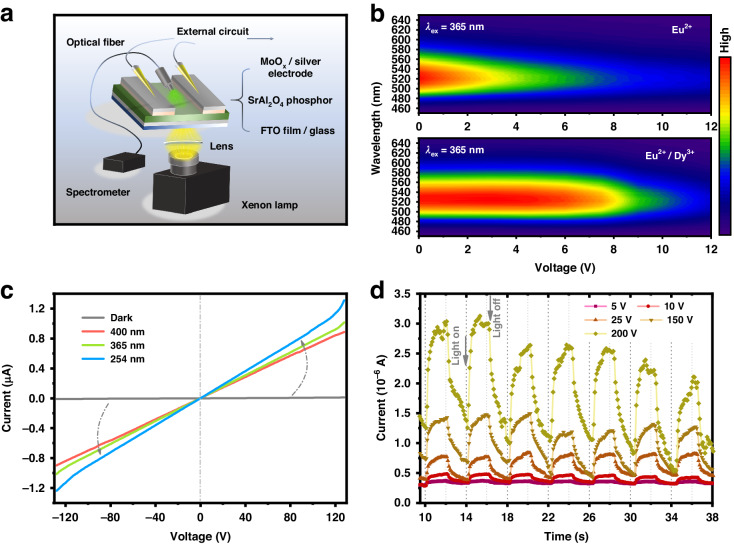
PL properties of SAO:Eu^2+^,Dy^3+^ under electrical stimulation. **a** Schematic diagram of fabricated devices applied for PL & PersL spectra under DC voltage. **b** DC voltage-dependent PL spectra of SAO:Eu^2+^ and SAO:Eu^2+^,Dy^3+^, activated wavelength is 365 nm. **c** The current-voltage relation of SAO:Eu^2+^,Dy^3+^ under different activated wavelengths. **d** The voltage-dependent photocurrent in SAO:Eu^2+^,Dy^3+^

First, single-doped SAO:Eu^2+^, co-doped Eu^2+^/Dy^3+^ (representing a shallow trap), and Eu^2+^/Yb^3+^ (representing a deep trap) were subject to PL spectral testing at a low DC voltage. Figure 2a presents the device schematic consisting of a MoO_x_/silver electrode, the SAO:Eu^2+^,RE^3+^ phosphor, and a transparent conductive FTO substrate. The SAO: Eu^2+^,Dy^3+^ layers are composed of closely contacted phosphor particles with a thickness of nearly 80 μm on average. The images of SAO:Eu^2+^,Dy^3+^ layers from cross-section and vertical view are shown in Fig. S8 and Fig. S9, where the constructed device could be placed into an external circuit for spectral measurements. By gradually increasing the DC voltage from 0 V to 12 V, the emission intensities of SAO:Eu^2+^,RE^3+^ decreased with increasing of full width at half maximum (FWHM), as summarized in Fig. 2b, Fig. S10, and Fig. S11. More specifically, the time-resolved photoluminescence (TRPL) spectrum of the above SAO:Eu^2+^,Dy^3+^ is exhibited in Fig. S12; here, a single emission band with a maximum intensity at 520 nm was observed, and this result was consistent with the former PL results. $\tau$ gradually decreases from 593.12 ns to 329.06 ns when a DC voltage is applied and can be calculated by the following^40,41^:

1 $$\frac{1}{\tau }=\frac{1}{{\tau }_{0}}+{A}_{{\rm{nr}}}+{P}_{{\rm{t}}}$$

where $\tau$ is the average decay lifetime, ${\tau }_{0}$ is the time component of the radiative transition, and ${A}_{{\rm{nr}}}$ and ${P}_{{\rm{t}}}$ are the components of nonradiative phonon relaxation and energy transfer, respectively. The decrease in the decay lifetime can be attributed to the increase in the nonradiative process of the Eu^2+^ 5d levels caused by an external voltage. The excited state of Eu^2+^ is located below the CBM, can be treated as an ultra-shallow state, and is easily stimulated by an external electric field via Coulomb interactions; thus, an infinitesimal disturbance leads to a slight curvature change of the corresponding configuration coordination curve, resulting in the broadening of the emission spectra^42,43^.

Furthermore, to confirm the trapping properties during excitation, the *I*/*V* curves under different activated wavelengths of SAO:Eu^2+^,Dy^3+^ were tested. When the excitation source was turned off, the dark current of SAO:Eu^2+^,Dy^3+^ was close to zero, and the photocurrents of SAO:Eu^2+^,Dy^3+^ were 0.89 μA, 1.01 μA and 1.31 μA under the excitation of 400 nm, 365 nm, and 254 nm, respectively, as shown in Fig. 2c. Applying converted bias also follows the same trends, this is because the phosphor layer is composed of polycrystalline SAO:Eu^2+^,Dy^3+^ powder, whose internal defects are statistically evenly distributed inside the lattice and exhibit isotropy under the macroscopic electric field stimulation^44^. Therefore, the current signal does not change significantly under a reversed bias. The variation in photocurrents could be attributed to photoionization under the excitation of high-energy photons, which could increase the density of carriers in the conduction band and the photocurrent. Figure 2d shows the voltage-dependent photocurrent of SAO:Eu^2+^,Dy^3+^ under 365 nm excitation, where the voltage applied ranged from 5 V to 200 V. As the applied bias continually increased to 200 V, the photocurrent signal was enhanced from ~0.2 μA to ~1.75 μA, which demonstrated that the electric field could drive the photogenerated electrons in the conduction band of SAO: Eu^2+^, Dy^3+^. The addition of Dy^3+^ could cause new shallow electron traps to form in the SAO host, thus, the photocurrent was enhanced compared with that of SAO:Eu^2+^; moreover, the intensity of the long afterglow emission was also enhanced at the same time, as confirmed in Fig. S13 and Fig. S14. Considering the above results, the photoionization electrons could be generated under UV pre-irradiation and were easily stimulated by an electric field; these results support the transfer of electrons through the conduction band under a light source^45–47^. Moreover, the study of Ueda et al. can complement the results, that is the afterglow of SAO:Eu^2+^,Dy^3+^ generated by 450 nm charging can be attributed to tunneling effects^48^. However, after 365 nm irradiation, the carriers move through the conduction band during the afterglow of SAO:Eu^2+^,Dy^3+^, where an external electric field can help generate electrons in the conduction band from stimulation in the trap centers.

**Fig. 3:**
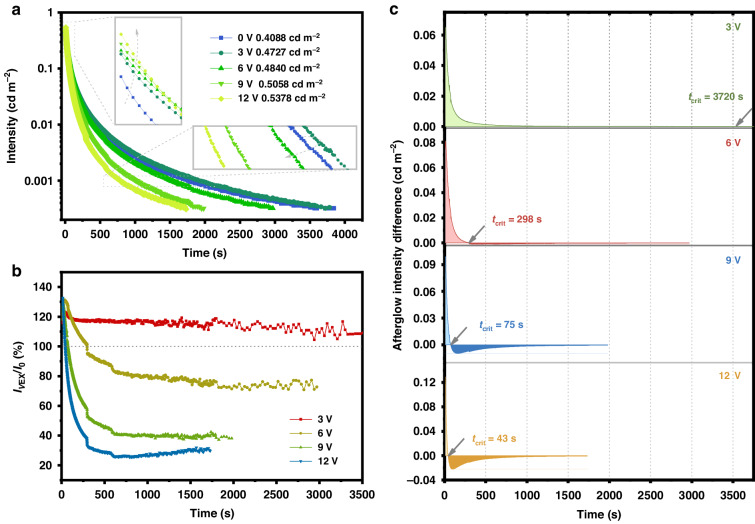
PersL properties of SAO:Eu^2+^,Dy^3+^ under electrical stimulation. **a** The DC electric field dependent PersL duration curves of SAO:Eu^2+^,Dy^3+^. **b** Comparison of afterglow brightness of SAO:Eu^2+^,Dy^3+^ with or without voltage stimulation. **c** The relationship between the difference in afterglow intensity and the DC voltage at different decay times

Subsequently, long afterglow duration curves were developed using a low DC electric field, as depicted in Fig. 3a and Fig. S15. With the increase in voltage to 12 V, the initial brightness of SAO:Eu^2+^,Dy^3+^ increases from 0.409 cd m^−2^ to 0.538 cd m^−2^ (increased by ~30%), while the afterglow duration decreases from 3840 s to 1730 s. These results indicate that after stable charging, the electrons captured by shallow traps can be rapidly released under the effect of the electric field, leading to the promotion of initial brightness. In Fig. 3b, the performance enhancement displays as the fraction between the afterglow intensity with and without voltage (*I*_*V*Ex_/*I*_0_) to better show the effective stimulation effect of 3 V and 6 V voltage. Through the evaluation of the difference in afterglow intensity in Fig. 3c, a critical time (*t*_crit_) is defined as the time when the afterglow brightness begins to decrease, and the infimum of afterglow brightness is specified as 0.32 mcd m^−2^ ^49,50^. The initial brightness of the afterglow is enhanced with the increase of the voltage, however, when the voltage gradually reaches 9 V, the brightness reduces rapidly. When the applied voltage is 3 V, the *t*_crit_ can reach 3720 s due to the positive electrical stimulation. As the voltage increases to 12 V, the afterglow quickly decreases and *t*_crit_ shrinks to only 43 s. This result indicates that the long afterglow duration stimulated by the electric field is mainly caused by the trap centers. This new phenomenon potentially occurs because the electrons trapped by shallow traps are more quickly released at higher voltage^51–53^. The mechanism is further substantiated next.

**Fig. 4:**
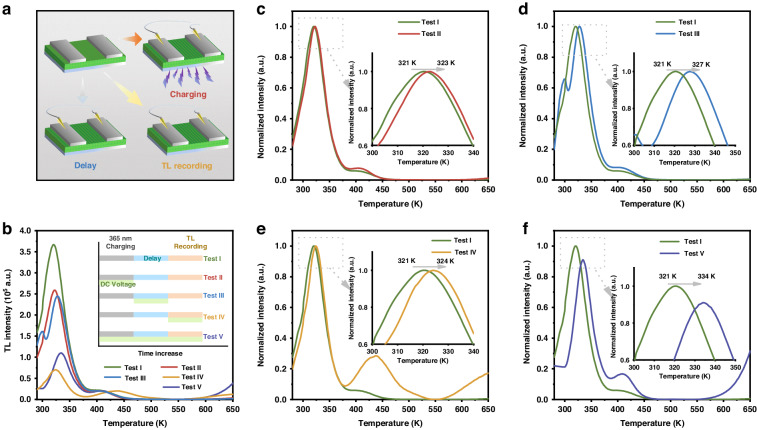
TL measurements of SAO:Eu^2+^,Dy^3+^ under electrical stimulation and the normalized TL glow curves in different voltage-applied conditions. **a** Scheme for applying voltage to SAO:Eu^2+^,Dy^3+^ during the TL test. **b** The TL glow curves of the SAO:Eu^2+^,Dy^3+^ sample in different voltage applied conditions. Inset is the schematic diagram of voltage-dependent TL measurement. **c** Applied a 12 V voltage at 10 s UV(365 nm) charging stage. **d** Applied a 12 V voltage at 10 s delaying stage. **e** Applied a 12 V voltage at the TL recording stage. **f** Applied a 12 V voltage at the above three stages

To further investigate the cause, the electric field-related TL glow curves of SAO:Eu^2+^,Dy^3+^ were arranged into three portions and are shown in Fig. 4a, b. All samples were heated at 673 K for 3 min for trap cleaning and then applied voltage sequentially in three processes: (1) charging at 365 nm for 10 s, (2) delay of 10 s, and (3) TL recording. TL glow Curve I was tested as a comparison.

1. At the pre-charging step (test II), the results are shown in Fig. 4c; here, the TL glow curve I features a double broad peak ranging from 300 K to 450 K, with maximums at 321 K and 406 K and depths of ~0.640 eV and 0.810 eV, respectively. Compared with curve I, the TL peak of test II moves to 323 K, and the signal in the low-temperature region is slightly reduced, this indicates that the electrons in the shallow trap are affected by the voltage.
2. At the delaying step (test III), as shown in Fig. 4d, the TL peak shifts from 321 K to 327 K as the intensity further decreases in the range from 330 K to 370 K. Additionally, the TL peak at 406 K remains stable.
3. At the recording step (test IV), the TL peak shifts from 321 K to 324 K in Fig. 4e, and the TL intensity decreases most while the peak intensity higher than 400 K is unchanged, which is consistent with the above. It should be pointed out that when the voltage is applied in all three processes, the test V in Fig. 4f, there is a similar change, but the peak position shifts the most, from 321 K to 334 K.

The variation in the rate-determining steps during long afterglow charging and discharging can be attributed to the following phenomenon^54,55^. When voltage is applied during the 365 nm pre-charging state, the photoionization of Eu^2+^ generates a large number of electrons in the conduction band; these electrons can be trapped and subsequently ensure the TL intensity. In contrast, when the excitation source is removed, the electric field stimulates the trap’s release at room temperature, which greatly aids in the return of the charge carriers from trap centers to the emission center; therefore, the TL peak appears at a higher temperature^56,57^. Additionally, a change in long afterglow emission is observed, and the external electric field can increase the return of the electrons to the conduction band, especially for those trapped in shallow regions. The shallow donor caused by Dy^3+^ is susceptible to the effect of an electric field, while the deep donor is often stable at a low DC electric field.

### Simulation of the electrically stimulated PersL

**Fig. 5:**
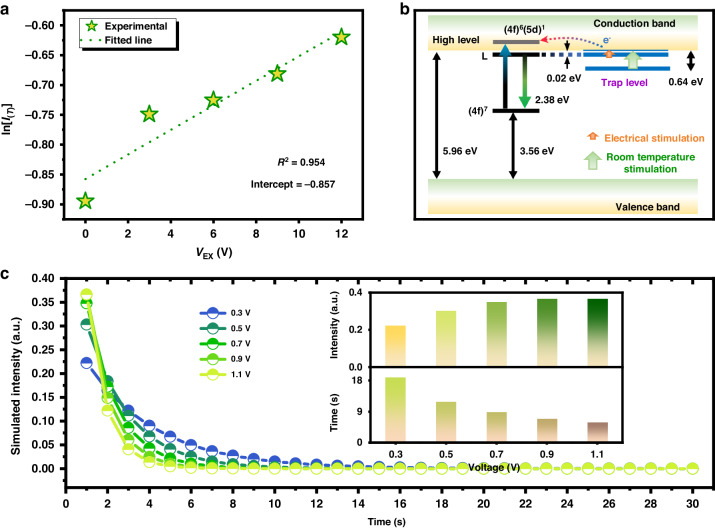
Simulation of the electrically stimulated PersL. **a** The linear fitted relation between ln [*I*_(*T*)_] and *V*_Ex_ of SAO:Eu^2+^,Dy^3+^. **b** The schematic electrically stimulated afterglow mechanism. **c** Simulation of afterglow decay curves under different voltages. Insets are the simulated initial brightness and the afterglow duration time when it decays to an intensity of 0.001

The TL glow intensity roughly satisfies the Arrhenius formula^58^,

2 $${I}_{(T)}=A\exp \left\{-\frac{\left(D\right)}{{KT}}\right\}$$

where ${I}_{(T)}$ is the intensity of the initial afterglow, $A$ is the pre-exponential factor, $D$ is the trap depth (eV), $K$ is the Boltzmann constant (1.38$\times$10^−23^ J K^−1^), and $T$ is the room temperature. At ambient temperature, the intensity TL is equivalent to the afterglow intensity released at room temperature. When an electric field is applied, the trap depth $D$ remains fixed (intrinsic property of the material), however, the additional electric field stimulates the electrons to jump from the shallow trap, giving them kinetic energy. Therefore, the formula can be approximated as follows:

3 $${I}_{(T)}\propto A\exp \left\{-\frac{\left(D-{V}_{{\rm{Ex}}}\right)}{{KT}}\right\}$$

where ${V}_{{\rm{Ex}}}$ is the external electric potential barrier. Conversely, in the case of long afterglow involving a relatively deep trap center, this caused by the external electric field is considered to be negligible^59^. Fig. 5a shows the well-fitted relationship between the intensity of the initial afterglow and the external electric voltage, and the ultra-shallow trap depth with voltage sensitivity is estimated to be 0.022 eV. These results are consistent with the reported works, where the lowest 5d energy level of Eu^2+^ in SAO corresponds to the conduction band with an energy difference of 20 meV (Ueda et al.) and 0.017 eV (Bos), respectively^48,60^. A possible mechanism is shown in Fig. 5b. An external electric field can significantly assist the jump of the trapped electrons from the ultra-shallow trap center to the conduction band at room temperature^61^. However, on the other hand, a higher voltage (12 V) induces the total number of trapped electrons to be released resulting in a rapid decrease in the subsequent afterglow brightness and afterglow duration. An external electric field occurs in a direction opposite to that of Coulomb force between metastable trap level and electrons outside the trap center in the conduction band; the reaction can be written as (Dy^3+^+e´)*→Dy^3+^+e´ (Releasing process, * refers to a metastable state), which allows electrons to stray in a direction opposite to the 5d state of Eu^2+^, partly resulting in the non-emissive process. This Coulomb force originated from the europium-trapped exciton^62–64^.

To demonstrate the dynamics of the afterglow process under a certain electric field, the rate equation describing the temporal evolution of electrons in the trap can be established. At first, consider the PersL process, the energy trap can be filled during the irradiation; however, the trapped electrons can also escape from the traps due to the stimulation of visible light and thermal stimulation of ambient temperature. The competition between trapping and de-trapping process is as follows:

4 $$\frac{{dN}}{dt}={{cP}}_{{\rm{Tr}}}n+\left(-c{P}_{{\rm{dTr}}}N\right)$$

where c is the coefficient, ${P}_{{\rm{Tr}}}$ is the trapping rate, ${P}_{{\rm{dTr}}}$ is the releasing or de-trapping rate, $N$ is the electron population in the trapped state, $n$ is the electron population in the excited state, and “$-$” indicates a decrease in the population of electrons trapped per second. The above equation is established based on the following assumptions: (1) Trap filling occurs continuously under the excitation photons; (2) The trapping or de-trapping rate is much smaller than the electron transition of the activator; (3) The influence of re-trapping on the electron population in the trap is ignored^65^. When the excitation source is removed, i.e., the afterglow emission process, the above equation can be transformed into:

5 $$\frac{{dN}}{dt}=-c{P}_{{\rm{dTr}}}N$$

since the trapping or de-trapping rate is fixed in the same PersLMs that depends on the nature of the intrinsic defects, ${P}_{{\rm{Tr}}}=-{P}_{{\rm{dTr}}}$ holds. The electric field stimulates the electrons to jump from the trap, giving them kinetic energy. This energy can be represented as a factor after a voltage stimulation. The formula can then be simplified:

6 $$-\frac{{dN}}{dt}=c{V}_{{\rm{Ex}}}{P}_{{\rm{Tr}}}N$$

When no voltage is applied, the afterglow duration can be expressed as ${I}_{\left(t\right)}\propto {P}_{{\rm{Tr}}}N$, and after taking the logarithm of both sides, that is $\mathrm{ln}{I}_{\left(t\right)}\propto \mathrm{ln}{P}_{{\rm{Tr}}}N$. As a voltage applied, the inverse of the afterglow intensity (*I*^−1^) as a function of time (t) can be obtained in Fig. S16, i.e., at 6 V, the *I*^−^^1^ ~ *t* curve ranging from 10 to 600 s can be effectively fitted to a straight line (*I*^−1^ = 0.199*t*–5.733), and the 600–3000 s region can be fitted to another straight line (*I*^−^^1^ = 1.093*t*–478.706); these correlate to fast decay and slow decay processes, respectively. The *I*^−1^ ~ *t* linear fitting results for the other voltages also yield a goodness-of-fit statistic (the parameters are listed in Table S5); a higher voltage correlates to a greater slope of the linear fitting. The linear fitting relationship between the afterglow intensity and the external voltage can be derived, namely:

7 $$\mathrm{ln}{I}_{\left(t\right)}\propto \mathrm{ln}{P}_{{\rm{Tr}}}N=-c{V}_{{\rm{Ex}}}t+{c}_{0}$$

where ${c}_{0}$ is a parameter related to the test conditions. When the illumination conditions and ambient temperature are the same, Eq. (2) can be transformed into the following exponential function:

8 $${P}_{{\rm{Tr}}}N\propto {c}_{0}\exp \left\{-c{V}_{{\rm{Ex}}}t\right\}$$

If the effect of de-trapping is not notable, Eqs. (3) and (5) can be combined to obtain the formula for the afterglow intensity with decay time under the influence of an external voltage, that is:

9 $${I}_{\left(t\right)}=-\frac{{dN}}{dt}\propto c{V}_{{\rm{Ex}}}\exp \left\{-{{cV}}_{{\rm{Ex}}}t\right\}$$

To determine the enhancement of afterglow under an external voltage, Eqs. (3) and (9) can be used as a guideline. This simple equation accurately simulates the decay rate of the afterglow intensity over time at a specific voltage, as illustrated in Fig. 5c. The observed decay trends in Fig. 3a support the credibility of the formula to some degree.

**Fig. 6:**
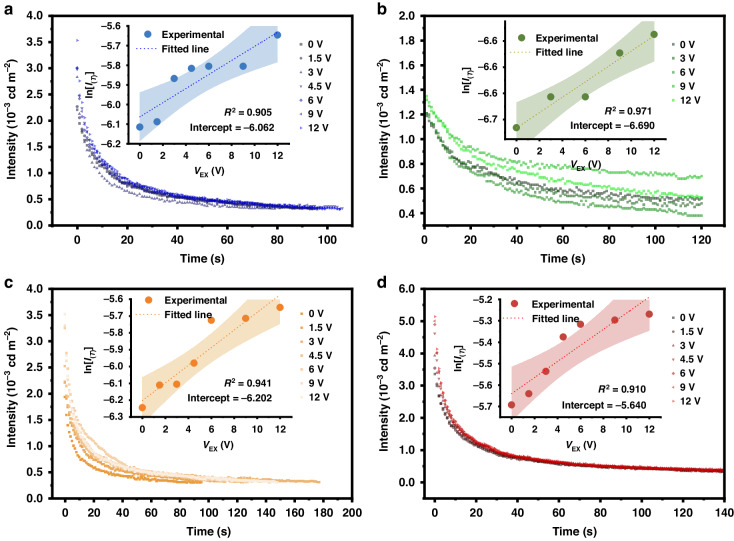
The voltage-dependent PersL duration. **a** CaAl_2_O_4_:Eu^2+^,Nd^3+^. **b** Ca_6_BaP_4_O_17_:Eu^2+^,Ho^3+^. **c** ZnS:Mn^2+^. **d** Y_2_O_2_S:Eu^3+^. The insets are linear fitted results between ln [*I*_(*T*)_] and *V*_Ex_ of the phosphors and the shaded areas represent 95% confidence intervals

To verify the generality of the design, several well-reported long afterglow materials were selected for the test. Figure 6 shows the correlations between the afterglow decay curves and applied voltage for samples CaAl_2_O_4_:Eu^2+^,Nd^3+^, Ca_6_BaP_4_O_17_:Eu^2+^,Ho^3+^, ZnS:Mn^2+^, and Y_2_O_2_S:Eu^3+^, respectively. The patterns are the same as those in Fig. 3a and Fig. S17, where the initial brightness of the afterglow was enhanced by applying a voltage up to 6 V for ~36% (0.00221 cd m^−2^ to 0.00301 cd m^−2^), ~15% (0.00124 cd m^−2^ to 0.00143 cd m^−2^), ~30% (0.00194 cd m^−2^ to 0.00253 cd m^−2^), and ~45% (0.00337 cd m^−2^ to 0.00491 cd m^−2^). Moreover, the good linear fitting results in the insets were similar to those in Fig. 5a, and the applied voltage extrapolation of the shallow trap depths for the stimulation of voltage could be obtained and are listed in Table 1. The trap depth of Ca_6_BaP_4_O_17_: Eu^2+^, Ho^3+^ is deeper, reaching 0.173 eV, which is why the initial brightness of the afterglow is not greatly enhanced. However, when the trap depth of Y_2_O_2_S:Eu^3+^ is shallower (0.146 eV), it is more susceptible to the electric field, which subsequently produces a greater increase. The peaks in the TL glow curves in Fig. S18 are located at 336 K, 357 K, 330 K, and 328 K, which also effectively corroborates the above analysis. In summary, the ultra-shallow traps in SAO:Eu^2+^,Dy^3+^ ensure efficient trap charging and release of carriers and produce enhanced afterglow brightness at low DC fields.

**Table 1:** The results of shallow trap depth extrapolated by the external voltage of different PersL phosphors

Phosphors	${\rm{D}}$ (eV)
SAO:Eu^2+^,Dy^3+^	0.022
CaAl_2_O_4_:Eu^2+^,Nd^3+^	0.171
Ca_6_BaP_4_O_17_:Eu^2+^,Ho^3+^	0.173
ZnS:Mn^2+^	0.161
Y_2_O_2_S:Eu^3+^	0.146

### Application of electrically stimulated PersL

**Fig. 7:**
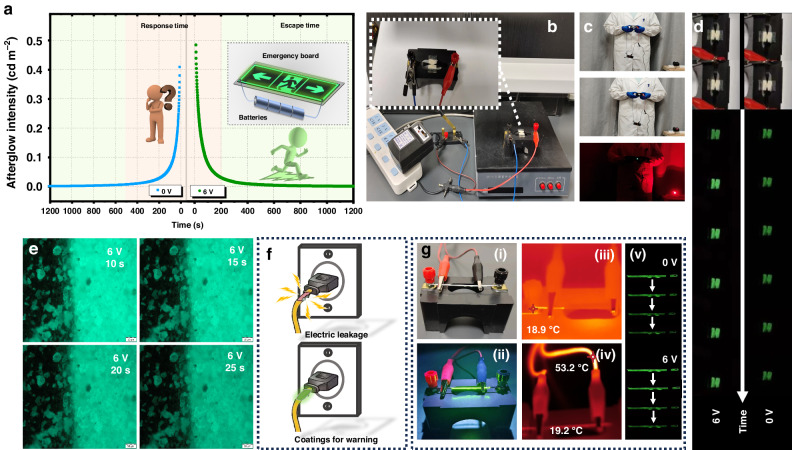
Application demonstrations of electrically stimulated PersL. **a** The long afterglow duration curves applied with 6 V voltage, and its schematic diagram in emergency escape, and inset is the schematic diagram of a voltage stimulated emergency board embedded with persistent phosphor. **b** The actual simplified circuit of devices applied an electric field stimulated afterglow emission. **c** A bright indicator of afterglow stimulated by the electric field. **d** Comparison of the afterglow intensity under 6 V voltage at different times. **e** The fluorescence microscope images of the interfaces between phosphors and electrodes. **f** An application demonstration of warning coatings for electrical leakage. **g** The photographs of energized wire with coatings: (i) under a fluorescent lamp, (ii) under UV light, (iii) infrared camera image (0 V), (iv) infrared camera image (6 V), (v) Afterglow brightness comparison of the coatings

As a consequence, Fig. 7a depicts a typical intelligent application based on the properties of long afterglow stimulated by an electric field. In the event of an unforeseen emergency and power outage in a building, a sign equipped with persistent phosphor can be powered by four dry batteries (6 V) to provide a brighter indication with minimal voltage needed. The simplified circuit shown in Fig. 7b is constructed and consists of a switch, a 6 V DC power source, a conductive adhesive, and a phosphor layer (uniformly coated on FTO). Since the influence of a low DC can increase the initial brightness by ~30% when the afterglow duration time is longer than 3000 s, this approach can provide quick warning signs to shorten the response time and greatly extend people’s escape time from disasters. As an example of this beneficial design, the bright PersL emission remains still visible at a distance of 5 meters from the observer in the darkroom, as shown in Fig. 7c. Additionally, Fig. 7d, e, Supporting Movie 1, and Supporting Movie 2 show the changes in the PersL brightness from the devices and submicron phosphor particles, where the voltage is applied, the afterglow brightness increases, and then the brightness gradually decreases. Another application demonstration is provided in Fig. 7f. For instance, electrical equipment can often cause serious accidents due to electricity leakage. However, by applying a coating prepared with PersLMs, an early warning can be achieved. As exhibited in Fig. 7g, the aluminum bare wire is coated with an afterglow coating (composed of SAO:Eu^2+^,Dy^3+^ and polymers). When a 6 V voltage is applied, a bright afterglow warning signal can be obtained before the wire temperature increases significantly, greatly improving safety.

## Discussion

In summary, an in-situ external electric field technique is proposed that combines phosphor/electrode structures to explore the afterglow variation and understand the mechanism of persistent phosphor SrAl_2_O_4_:1%Eu^2+^,2%RE^3+^ (SAO:Eu^2+^,RE^3+^) from an experimental standpoint. The research findings indicate that an external electric field can lead to a broadening of the PL peaks and a reduction in intensity. The afterglow duration curves provide compelling evidence for the stimulation of shallow traps by an external electric field. This phenomenon is estimated through both the Arrhenius and rate equations, which reveal a trap depth of 0.022 eV and a consequent 30% improvement in the initial brightness (0.538 cd m^−2^). Similar trends are observed for several existing persistent phosphors; these include CaAl_2_O_4_:Eu^2+^,Nd^3+^, Ca_6_BaP_4_O_17_:Eu^2+^,Ho^3+^, ZnS:Mn^2+^, and Y_2_O_2_S:Eu^3+^, with an increase of 36%, 15%, 30%, and 45%, respectively. The thermoluminescence (TL) peak, which is dependent on voltage, shifts from 321 K to a higher temperature, suggesting that the voltage facilitates the release of the trapped electrons.

We observed an “external electric field-stimulated enhancement of initial afterglow”. These findings reveal that electrons are transferred through the conduction band during the long afterglow charged by UV light; here, the rate-determining step for the electron from the trap center to the conduction band can be accelerated by an external electric field. The new design, consisting of SAO:Eu^2+^,RE^3+^ and using 6 V, provides a brighter emergency indicator than the previous design, which consisted of only SAO:Eu^2+^,RE^3+^. This study provides a strategy for studying lanthanide-doped persistent phosphors, which can trigger theoretical research and the exploration of functional materials with outstanding performance.

## Materials and methods

### Phosphor synthesis

SrAl_2_O_4_:1%Eu^2+^ and series SrAl_2_O_4_:1%Eu^2+^,2%RE^3+^ (Abbreviated as SAO:Eu^2+^,RE^3+^) samples were synthesized by simple solid-state reaction. Stoichiometrically weighted Sr_2_CO_3_(Macklin, A.R.), Al_2_O_3_(Macklin, A.R.), Eu_2_O_3_(Macklin, 5N), La_2_O_3_(Macklin, 4N), CeO_2_(Macklin, 4N), Pr_6_O_11_(Macklin, 4N), Nd_2_O_3_(Macklin, 4N), Sm_2_O_3_(Macklin, 4N), Gd_2_O_3_(Macklin, 4N), Tb_4_O_7_(Macklin, 4N), Dy_2_O_3_(Macklin, 4N), Ho_2_O_3_(Macklin, 4N), Er_2_O_3_(Macklin, 4N), Tm_2_O_3_(Macklin, 4N), Yb_2_O_3_(Macklin, 4N), and Lu_2_O_3_(Macklin, 4N) to the agate mortar respectively, then ground for 40 min. The mixture was sintered at 1573 K for 6 h under a reduction atmosphere (N_2_/H_2_ = 95/5, purity is 99.99%) in an alumina crucible. After cooling to room temperature, the powder was ground again for further characterization. Series SAO:RE^3+^ samples were also synthesized by simple solid-state reaction. After stoichiometrically weighted, and ground the raw materials, the mixture was sintered at 1573 K for 6 h under air.

### Phosphor-electrode structure fabrication

First mix the previously sintered SAO:Eu^2+^,RE^3+^ (0.5 g) with ethanol (0.02 ml), naphthol (0.01 ml) and terpineol (0.04 ml) as a solution. Then, ultra-sonicated the mixed solution for 15 min and stirred for 30 min. A coating layer (1 cm × 1 cm) of SAO:Eu^2+^,RE^3+^ phosphor with an average thickness of 80 μm is formed by the spin coating cycles 6 times on the conductive side of FTO-coated glass and dried at 60 °C for 2 h. Then using vacuum evaporation (0.5 × 10^−4 ^Pa), a MoO_x_ layer with a thickness of 80 nm was first coated, followed by a thin Ag layer with a thickness of 300 nm.

### Characterization

The crystal XRD data was obtained by Rigaku D/Max-2400 X-ray diffractometer (Cu Kα radiation, *λ* = 1.54178 Å, 2θ ranges from 10° to 80°). Based on standard phase data (26466-ICSD), the crystal structures were simulated by Rietveld structural refinement in the GSAS program and depicted by VESTA software. The surface morphology of SAO:Eu^2+^,RE^3+^ were taken by Hitachi S-3400 scanning electron microscope. Photoluminescence (PL) and photoluminescence excitation (PLE) spectra were examined by Horiba Fl3-21 fluorescence spectrometer. TRPL spectra were studied by EDINBURGH INSTRUMENTS FS5 spectrofluorometer. The cathodoluminescence (CL) properties of the samples were obtained using a modified Mp-Micro-S instrument. The diffuse reflectance spectra of SAO were measured by the Perkin Elmer 950 spectrometer at room temperature. Electron spin resonance spectra (ESR) were collected by JES-FA300 ESR spectrometer (Microwave Frequency is 9448.802 MHz, Power is 0.998000 mW) at room temperature. After heated at 673 K to clean the trapped carriers, the long persistent luminescence (PersL) decay curves were recorded by the PR305 afterglow photometer (Zhejiang University Sensing Instruments); and the TL spectra of SAO: Eu^2+^, RE^3+^ were acquired by aFJ-427A TL instrument (Beijing Nuclear Instrument Factory) and TOSL-3DS TL spectrometer (Guangzhou Radiation Technology Co., Ltd.), the heating rate is kept at 1 K s^−1^, charging at 365 nm for 10 s, more details can be found in Supporting Information (Tables S6 and S7). The photocurrent and electrochemical impedance spectroscopy were completed by CORRTEST (CS310) and Zahner Zennium electrochemical workstation, respectively. The voltage-current relation was recorded by the sensitive galvanometer. All the digital pictures were taken by the Canon EOS 4000D smart camera.

### Supplementary information

Supporting Information

Supporting Movie 1

Supporting Movie 2
