# Supplementary material for: Electrical stimulation for brighter persistent luminescence
Source: Light Sci Appl. 2024 Jul 15;13:165. doi: 10.1038/s41377-024-01507-0 (PMC11251151; doi:10.1038/s41377-024-01507-0)
Supplement: Supplementary file 1 — Supporting Information [file 41377_2024_1507_MOESM1_ESM.docx]

Supporting information for

**Electrical stimulation for brighter persistent luminescence**

*Xilin Ma ^a^, Yuhua Wang ^a,^***, Takatoshi Seto ^a,^**

*^a^* Key Laboratory for Special Function Materials and Structural Design of the Ministry of Education,

National & Local Joint Engineering Laboratory for Optical Conversion Materials and Technology of National Development and Reform Commission, Department of Materials Science,

School of Materials and Energy, Lanzhou University, No. 222, South Tianshui Road, Lanzhou, Gansu, 730000, P. R. China.

*Corresponding author’ email: Takatoshi Seto - E-mail: seto@lzu.edu.cn

Yuhua Wang - E-mail: wyh@lzu.edu.cn

ID 0000-0002-3928-2557 (T. Seto), ID 0000-0001-7047-4760 (Y. Wang)

Content

**Supplementary Figures** S4

Fig. S1 Statistics of some PLMs S4

Fig. S2 XRD patterns S5

Fig. S3 DR spectra S6

Fig. S4 PersL decay curves S7

Fig. S5 PersL performances and related TL spectra S8

Fig. S6 Photocurrent signal S10

Fig. S7 PL, PLE, and CL spectra S11

Fig. S8 SEM image of phosphor-electrode structure S12

Fig. S9 Fluorescence microscope images of phosphor-electrode structure S13

Fig. S10 Voltage-dependent PL spectra S14

Fig. S11 PL intensities comparison S15

Fig. S12 Voltage-dependent TRPL spectra S16

Fig. S13 EIS and ESR spectra S17

Fig. S14 A proper mechanism diagram at the atomic level S19

Fig. S15 Voltage-dependent PersL spectra S20

Fig. S16 Fitting curves of the afterglow (*I*^-1^) as a function of time S21

Fig. S17 TL spectra of the other four PLMs S22

Fig. S18 Comparison of afterglow brightness S23

**Supplementary Tables** S24

Table S1 Crystallographic parameters and Rietveld refinement results S24

Table S2 Refined atomic coordinates S24

Table S3 Afterglow brightness at different durations S24

Table S4 Trap depths of lanthanide ions doped SAO samples S25

Table S5 Fitting parameters of the afterglow (*I*^-1^) as a function of time S25

Table S6 Test conditions of thermoluminescence (TL) glow curves S25

Table S7 Test conditions of afterglow duration curves S25

**Reference S26**

**Supplementary Figures**

**
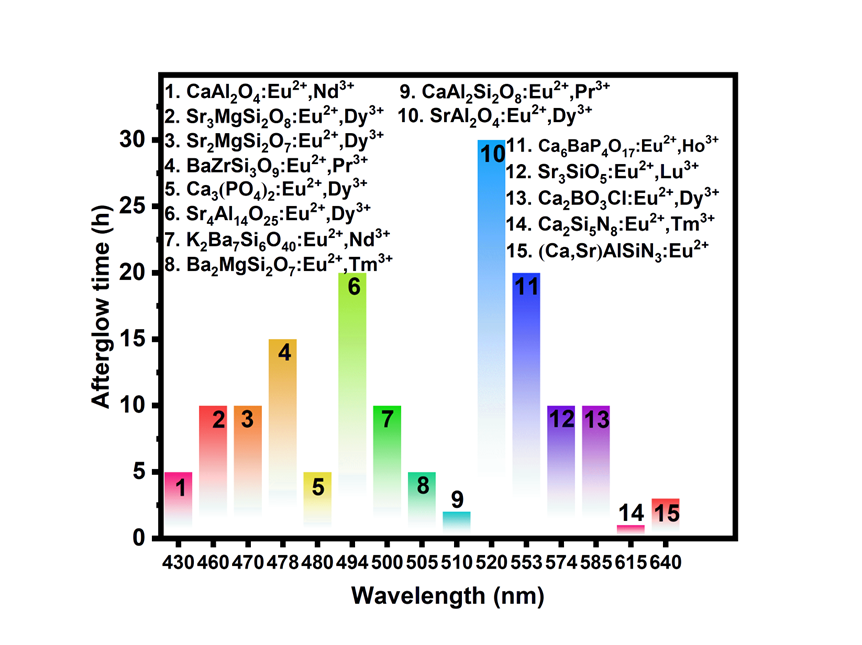
**

**Fig. S1** Some long afterglow materials with better properties. ^1-5^

**
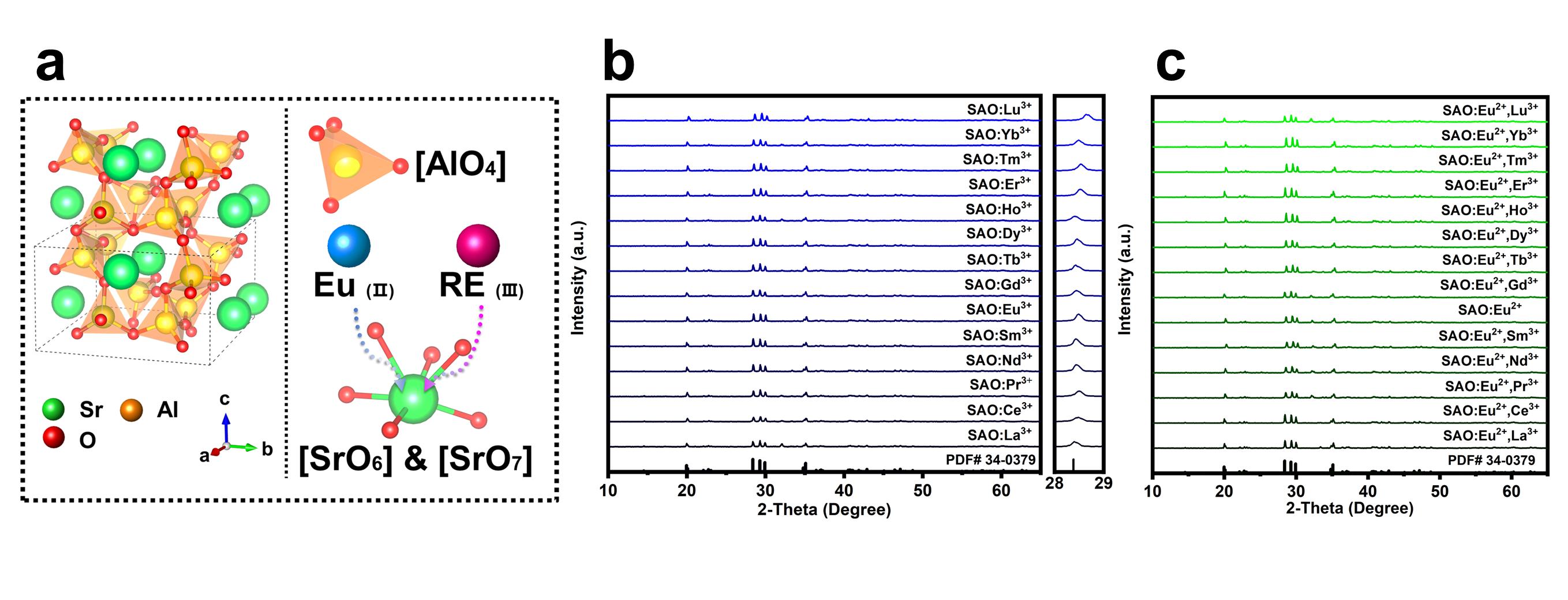
**

**Fig. S2** **a** Diagram of SAO crystal structure and cation coordination. **b** XRD patterns of single-doped SAO: RE^3+^. The right side is the peak shift of ($\bar{2}11$). **c** XRD patterns of SAO:Eu^2+^ and co-doped SAO:Eu^2+^,RE^3+^.

**Note:** Fig. S2a exhibits the crystal structure schematic diagram of the SAO host, where the [AlO_4_] polyhedron is connected by sharing the vertex, forming a six-membered ring, where the Sr atom is occupied, generating the coordination of [SrO_6_] or [SrO_7_] (usually differs in chemical bond length and local symmetry).^6,7^ Since the radius of lanthanide ions are close to Sr^2+^ [for instance, Sr^2+^ (CN=6, 1.18 Å, CN=7, 1.21 Å), Eu^3+^ (CN=6, 0.947 Å, CN=7, 1.01 Å)] which can provide suitable occupation sites for them to dope in SAO.^8^ As shown in Fig. S2b, SAO:RE^3+^ samples show no evident impurity stand-by indexation of PDF# 34-0379 as well as the crystal structure refinement results of SAO:Eu^2+^,Dy^3+^ in Fig. 1a. With the increase of the atomic number of the doped lanthanide ions, the diffraction peak ($\bar{2}11$) gradually moved to a higher angle, indicating that the crystal plane spacing is decreasing gradually. Fig. S2c reveals the same relation in co-doped SAO:Eu^2+^,RE^3+^.

**
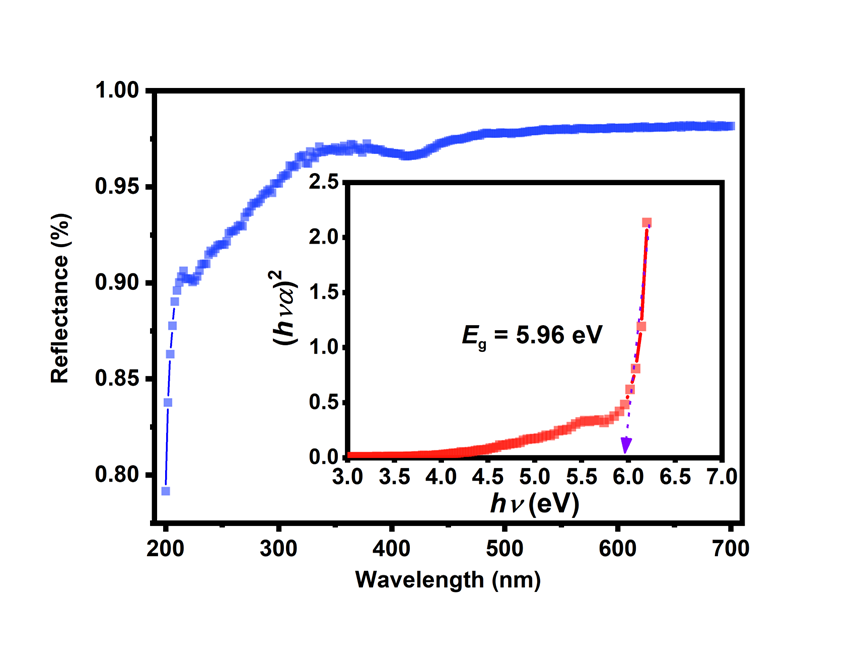
**

**Fig. S3** The DR spectrum of the SAO host sample. Inset is the straight-line extrapolation result of the SAO host sample by the Kubelka-Munk formula.

**Note:** On the basis of the diffuse reflectance (DR) spectrum, the optical band gap energy was obtained as 5.96 eV of SAO host shown in Fig. S3.^9,10^

**
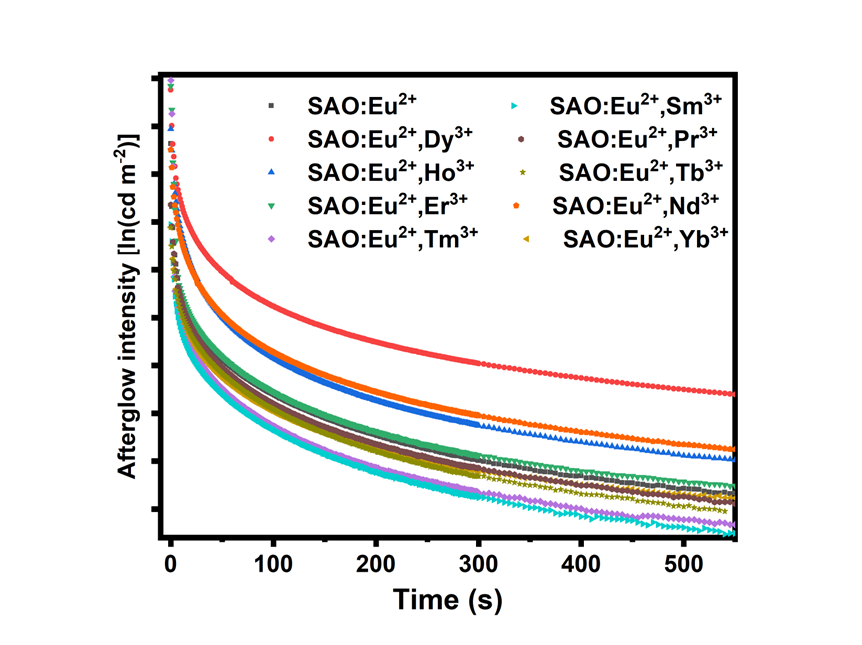
**

**Fig. S4** PersL decay curves of SAO:Eu^2+^ and co-doped SAO:Eu^2+^,RE^3+^ samples.


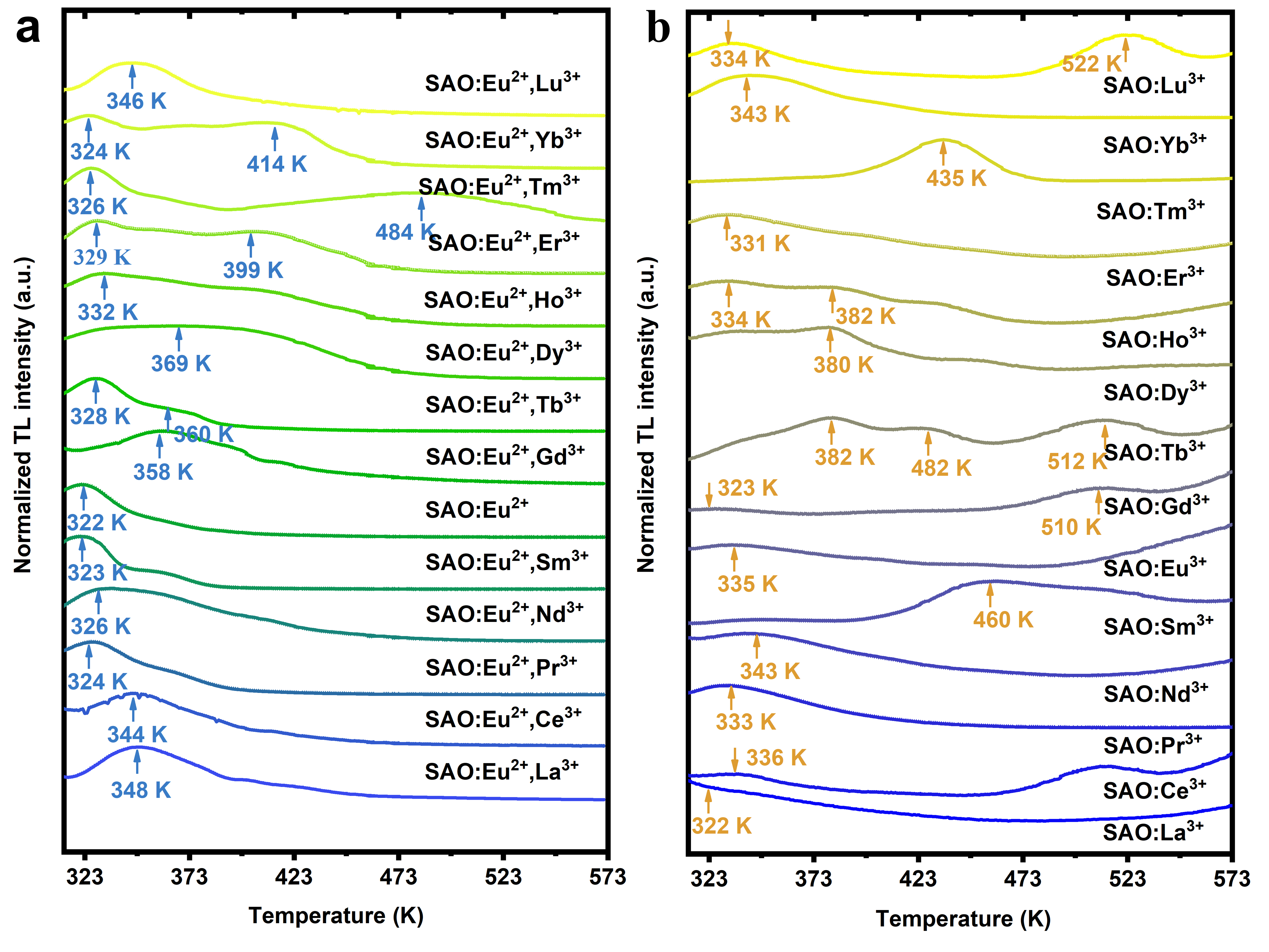


**Fig. S5** The TL glow curves of lanthanide ions doped SAO samples: **a** SAO:Eu^2+^ and SAO:Eu^2+^,RE^3+^, **b** SAO: RE^3+^.

**Note:** Based on the afterglow duration curves of SAO:Eu^2+^,RE^3+^ (Fig. S4), the TL spectra in Figs. S5a and S5b are systemically examined, and the trap depths can be correspondingly estimated by the following equation: ^11,12^

$E_{t}=\frac{T_{m}}{500}$ (1)

where $E_{t}$ is the depth of trap level, $T_{m}$ is the peak temperature of the TL curves. Co-doping with different divalent/trivalent lanthanide ions can introduce different traps into SAO: Eu^2+^, RE^3+^, and SAO:Eu^2+^,Dy^3+^ has appropriate trap depth (0.738 eV) and sufficient trap concentration acquires good afterglow. The Er^3+^, Tm^3+^, Yb^3+^, and Lu^3+^ co-doped samples have deep traps (0.798 eV, 0.812 eV, 0.968 eV, 0.834 eV, respectively) and are not conducive for long afterglow at room temperature. The co-doping of Ce^3+^, Pr^3+^, Nd^3+^, Sm^3+^, Tb^3+^, and Ho^3+^ oppositely have shallow trap levels (0.688 eV, 0.69 eV, 0.652 eV, 0.672 eV, 0.72 eV, 0.722 eV, respectively), due to the lower trap concentration, the afterglow durations are still short (include the La^3+^, Gd^3+^ co-doping).

When constructing the diagrams of VRBE and HRBE, the following are the steps:

(1) Based on the diffuse reflectance spectrum of the host, the optical band gap energy is 5.96 eV of SAO.

(2) Then the energy of charge transfer (*E*^CT^) can be obtained from UV activated spectrum of the Sm^3+^ doped sample, which is represented by the energy difference between Sm^2+^ ground state energy $E_{4f}\left( 6,2+,SAO \right)$ and the VBM. Similarly, *E*^CT^ can also be obtained from the band of charge transfer transition of the excitation spectrum of the Eu^3+^ doped sample.

(3) The difference between 4f-Ln^2+^ and 4f-Ln^3+^ largely depends on the hosts. However, it is relatively stable in condensed matter, often taking 6~7 eV in oxides, hence the $U\left( 6,A \right)$ takes 6.50 eV. ^13^

Further, the energy levels in the HRBE diagram need to be converted into absolute energy (relative to the Vacuum environment), that is to construct the VRBE diagram. Based on Dorenbos theory, the absolute energy of ground Eu^2+^ 4f state $E_{4f}\left( 7,2+,A \right)$, can be estimated under chemical shift model and listed below: ^14,15^

$E_{4f}\left( 7,2+,A \right)=E_{4f}\left( 7,2+,Vacuum \right)+\frac{18.05-U\left( 6,A \right)}{0.777-0.0353U\left( 6,A \right)}$ (S-1)

where, $E_{4f}\left( 7,2+,Vacuum \right)$ is a constant takes -24.92 eV. In this case, the absolute energies of ground Eu^2+^ 4f state, CBM and VBM are estimated to be -3.83 eV, -3.25 eV and -7.36 eV.

**
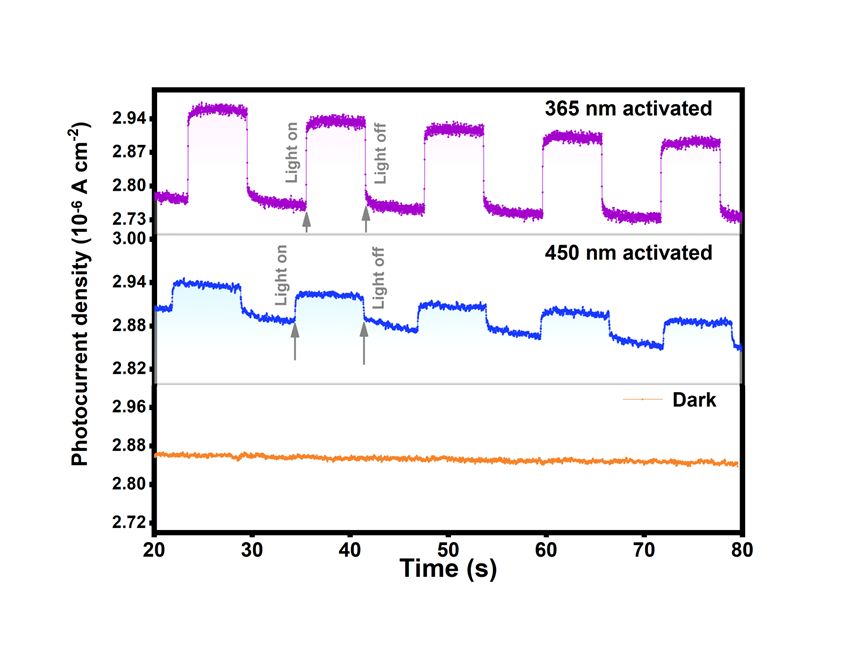
**

**Fig. S6** The photocurrent of SAO:Eu^2+^,Dy^3+^ under different activation conditions.

**Note:** The wavelength-dependent *I*-*V* relation of SAO:Eu^2+^,Dy^3+^ is measured, and described in Fig. S6, though SAO:Eu^2+^,Dy^3+^ shows no obvious current signal in the dark, the average photocurrent signal values reach ~0.05×10^-6^ A cm^-2^ and ~0.2×10^-6^ A cm^-2^ under 450 nm or 365 nm ultraviolet (UV) irradiated, respectively.

**
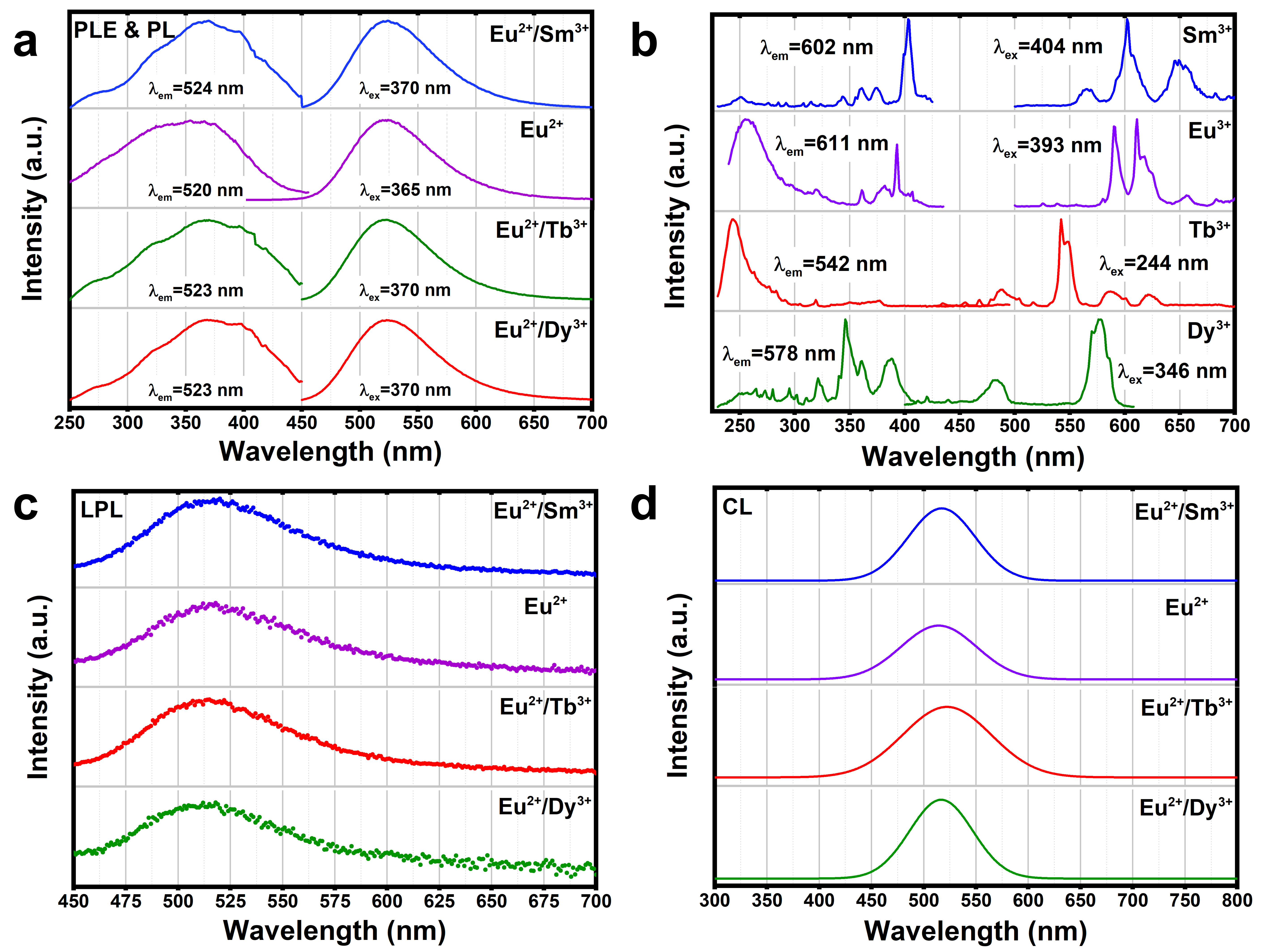
**

**Fig. S7** **a** PL and PLE spectra of single-doped SAO:Eu^2+^ and co-doped SAO:Eu^2+^,RE^3+^ samples (RE^3+^ = Sm^3+^, Tb^3+^, Dy^3+^). **b** PL and PLE spectra of SAO:RE^3+^ samples (RE^3+^ = Eu^3+^, Sm^3+^, Tb^3+^, Dy^3+^). **c** PersL spectra of single-doped SAO:Eu^2+^ and co-doped SAO:Eu^2+^,RE^3+^ samples (RE^3+^ = Sm^3+^, Tb^3+^, Dy^3+^). **d** CL spectra of single-doped SAO:Eu^2+^ and co-doped SAO:Eu^2+^,RE^3+^ samples (RE^3+^ = Sm^3+^, Tb^3+^, Dy^3+^).

**Note:** The PL and PLE spectra in Fig. S7a are not significantly changed after introducing Sm^3+^, Tb^3+^ and Dy^3+^ matched with Fig. 1b, and no obvious sharp peak emission of Ln^3+^ ions is observed (compared with Fig. S7b), as well as the PersL emission and cathode-ray luminescence (CL) spectra in Figs. S7c-d.

**
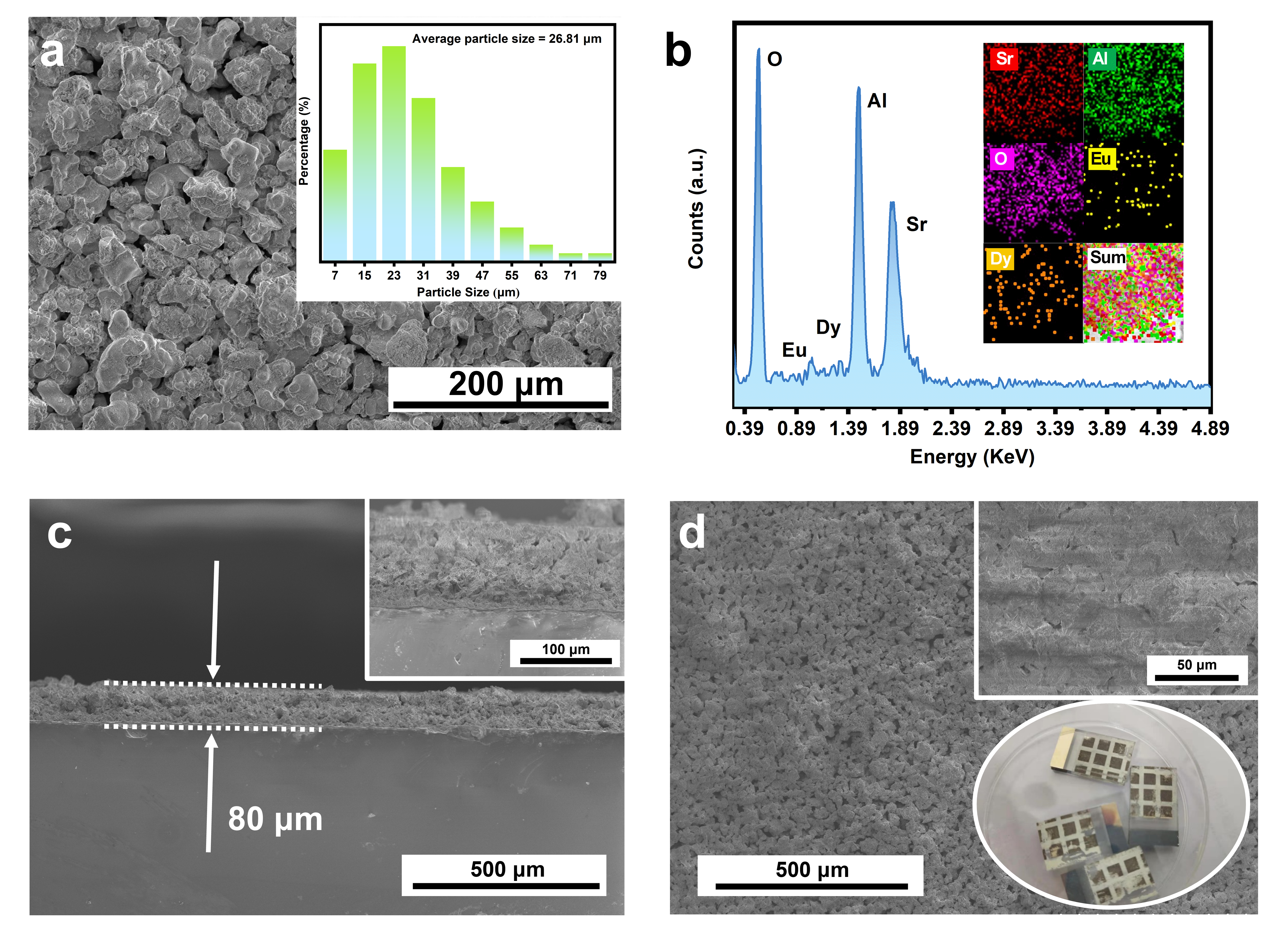
**

**Fig. S8** **a** SEM image of SAO: Eu^2+^, RE^3+^ sample applied in phosphor-electrode structure fabrication. Inset is the particle size distribution of SAO:Eu^2+^,Dy^3+^. **b** EDX spectrum of SAO:Eu^2+^,Dy^3+^. Inset is the mapping-scan images of SAO:Eu^2+^,Dy^3+^. **c** SEM images of SAO:Eu^2+^,Dy^3+^ layers from cross-section view. Inset is the locally zoomed image. **d** SEM images of SAO: Eu^2+^,Dy^3+^ layers from vertical view. Insets are the locally zoomed image and the photograph of fabricated devices.

**Note:** Fig. S8a shows the morphology of SAO:Eu^2+^,Dy^3+^, in which irregular particles own the rough surface, the particle size distribution of SAO:Eu^2+^,Dy^3+^ shows an average size of 26.81 μm. Moreover, the EDX spectra and mapping scan images in Fig. S8b of SAO:Eu^2+^,Dy^3+^ show the elemental composition of the sample is Sr, Al, O, Eu, and Dy elements and without other impurity elements. The results are consistent with the XRD analysis.

From a cross-section view, there is a uniformly coated thin layer on the substrate, with an estimated thickness of about 80 μm. The vertical view shows the phosphor particles are closely contacted in Figs. S8c and d.

**
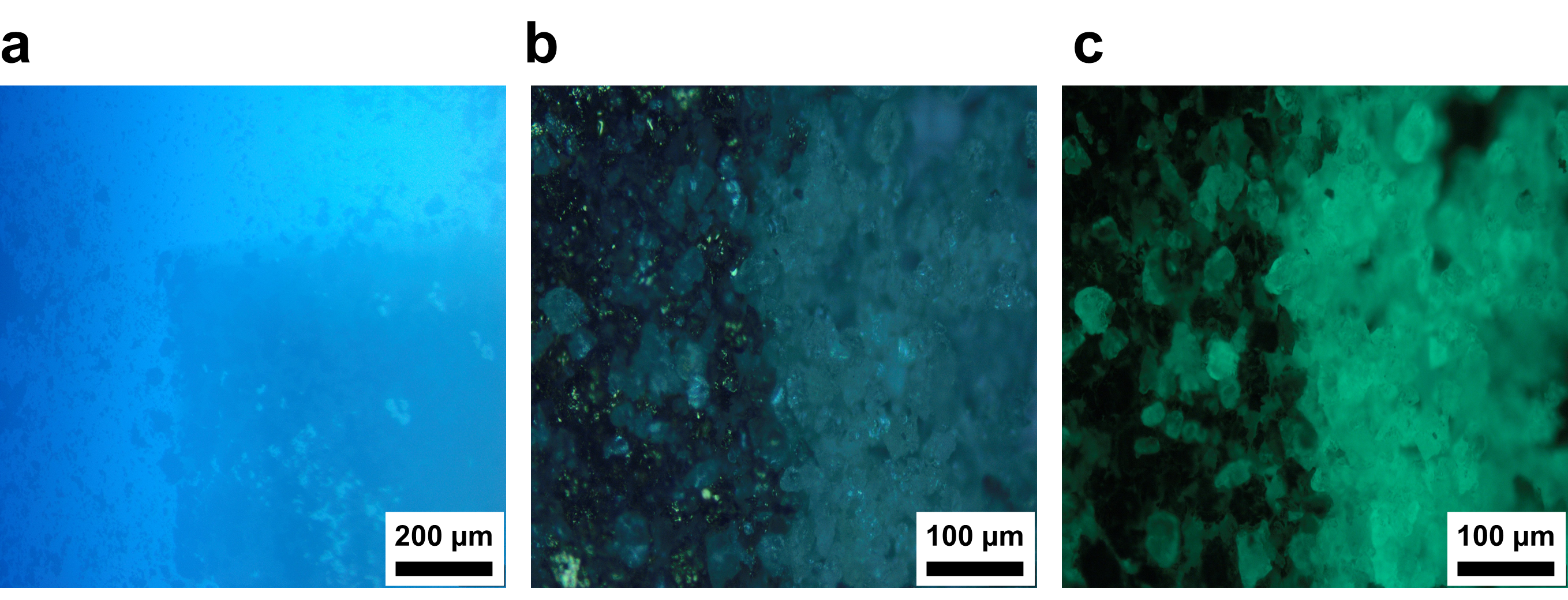
**

**Fig. S9** The fluorescence microscope images of SAO: Eu^2+^, Dy^3+^ sample applied in phosphor-electrode structure: **a** The bright field image, **b** The bright field image at high magnification, **c** The dark field image.

**Note:** As shown in Fig. S9a, the electrode boundary can be clearly distinguished. The contact interface of the electrode and SAO: Eu^2+^, Dy^3+^ at high magnification is also observed in Fig. S9b. The afterglow in the dark field is identifiable in Fig. S9c.

**
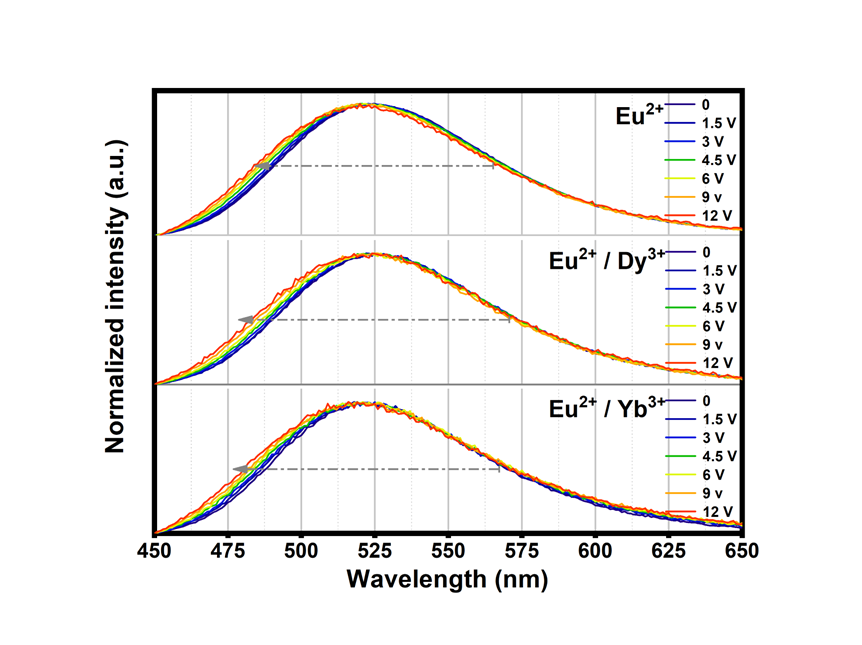
**

**Fig. S10** The voltage dependent PL spectra of single-doped SAO:Eu^2+^ and co-doped SAO:Eu^2+^,RE^3+^ (RE^3+^ = Dy^3+^, Yb^3+^), activated at 365 nm.


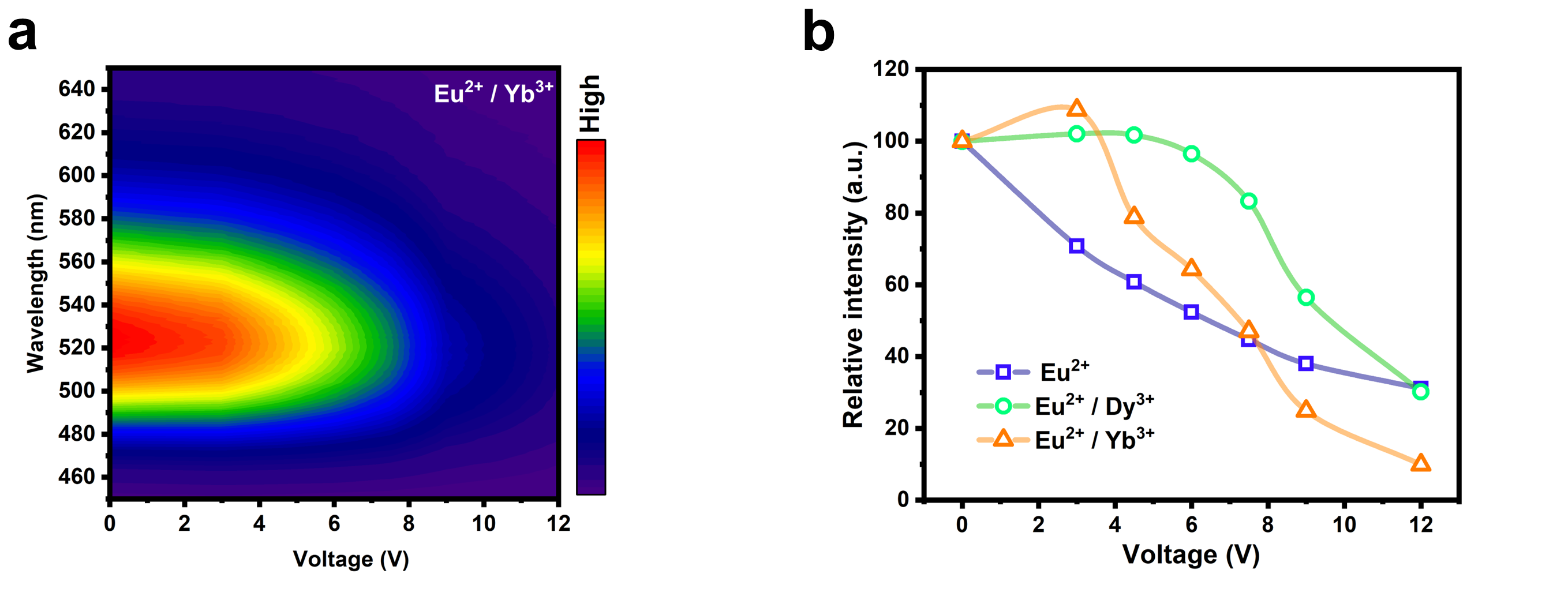


**Fig. S11** **a** The voltage-dependent PL spectra of co-doped SAO: Eu^2+^, Yb^3+^ are depicted in the 3D color mapping surface (activated at 365 nm). **b** The relationship between relative PL intensities in single-doped SAO:Eu^2+^ and co-doped SAO:Eu^2+^,RE^3+^ (RE^3+^ = Dy^3+^, Yb^3+^).

**
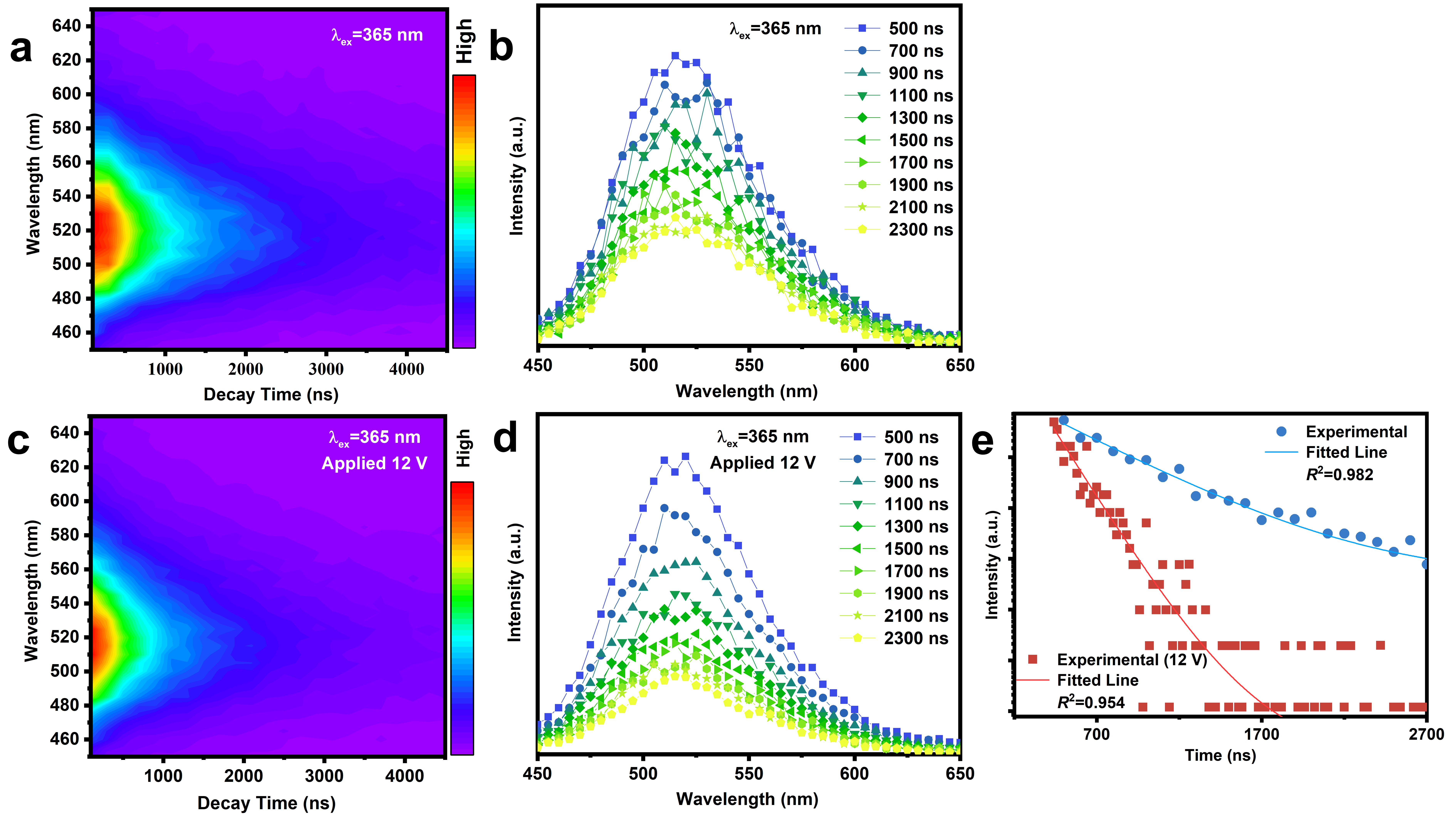
**

**Fig. S12** Voltage-dependent time-resolved PL (TRPL) spectra of SAO:Eu^2+^,Dy^3+^ sample activated at 400nm: **a** applied 0 V, **c** applied 12 V. And PL intensity over different decay lifetimes: **b** applied 0 V, **d** applied 12 V. **e** Decay curves of SAO:Eu^2+^,Dy^3+^ activated by 365 nm.


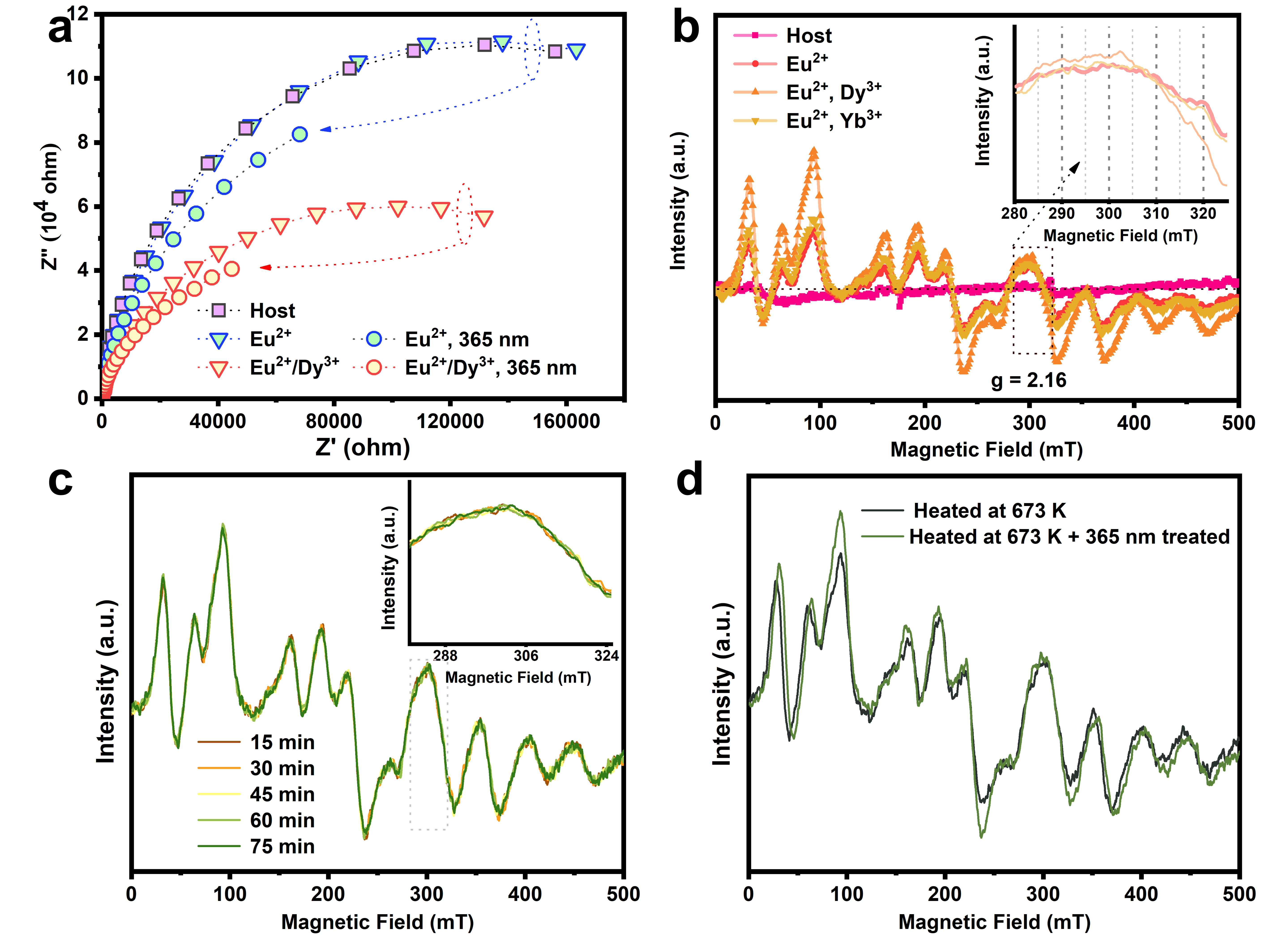


**Fig. S13** **a** EIS of SAO host, single-doped SAO:Eu^2+^ and co-doped SAO:Eu^2+^,RE^3+^ (RE^3+^ = Dy^3+^, Yb^3+^) under darkness and activated at 365 nm. **b** ESR spectra of SAO host, single-doped SAO:Eu^2+^ and co-doped SAO:Eu^2+^,RE^3+^ (RE^3+^ = Dy^3+^, Tm^3+^). Inset is local zoomed spectra. **c** The time-dependent ESR spectra of SAO:Eu^2+^,Dy^3+^. **d** ESR curves of SAO:Eu^2+^,Dy^3+^ after trap cleaning at 673K and charging at 365 nm for 10 s after trap cleaning.

**Note:** The SAO:Eu^2+^,Dy^3+^ under 365 nm irradiated owns the smallest radius of curvature of the electrochemical impedance spectroscopy (EIS) curves demonstrated in Fig. S13a, which implies that SAO: Eu^2+^, Dy^3+^ produces photogenerated carriers after absorbing the UV light at 365 nm.^16^ Fig. S13b compares the ESR spectra between the SAO: Eu^2+^, RE^3+^ and SAO host. Due to the 4f^7^ configuration of Eu^2+^, it has an orbital singlet ^8^S_7/2_ ground term. When the electron spin magnetic moment of Eu^2+^ interacts with the external magnetic field, considering two spin states the energy levels split (-7/2→-5/2, -5/2→-3/2, -3/2→-1/2, -1/2→1/2, 1/2→3/2, 3/2→5/2, 5/2→7/2), the resonant field corresponding to the zero value of the first differential ESR signal can be detected. The host sample lacks the ESR signal, but the signal appears after adding Eu^2+^, indicating that the ESR signals with fine structures originated from Eu^2+^. The relative height of g = 2.16 peak is Eu^2+^/Dy^3+^ > Eu^2+^/Yb^3+^ > Eu^2+^, which declares that the increased signal of unpaired electrons is largely generated via Dy^3+^ co-doped (formed as electron traps, which can bound free electrons). ^17,18^

Then the time-dependent ESR experiment was carried out. The SAO:Eu^2+^,Dy^3+^ was first exposed to visible light for 15 minutes, and then the ESR measurement began. Following completion of the test, the exposure to ambient light was continued for an additional 15 minutes, and each data group was realized in this way. As depicted in Fig. S13c, there were no significant changes in the ESR signals over time.

The effect of 365 nm irradiation is also confirmed. As can be seen in Fig. S13d, there is no significant change in the shape of the ESR spectra before and after 365 nm irradiation. Only the intensity becomes stronger, which can indicate that after 365 nm irradiation, more electrons are trapped by the traps, and the unpaired electrons increase. Compared with the original SAO host sample, it can be concluded that there is a higher concentration of electron traps in SAO:Eu^2+^,Dy^3+^, that is, the introduction of Dy^3+^ increases the concentration of electron traps.

**
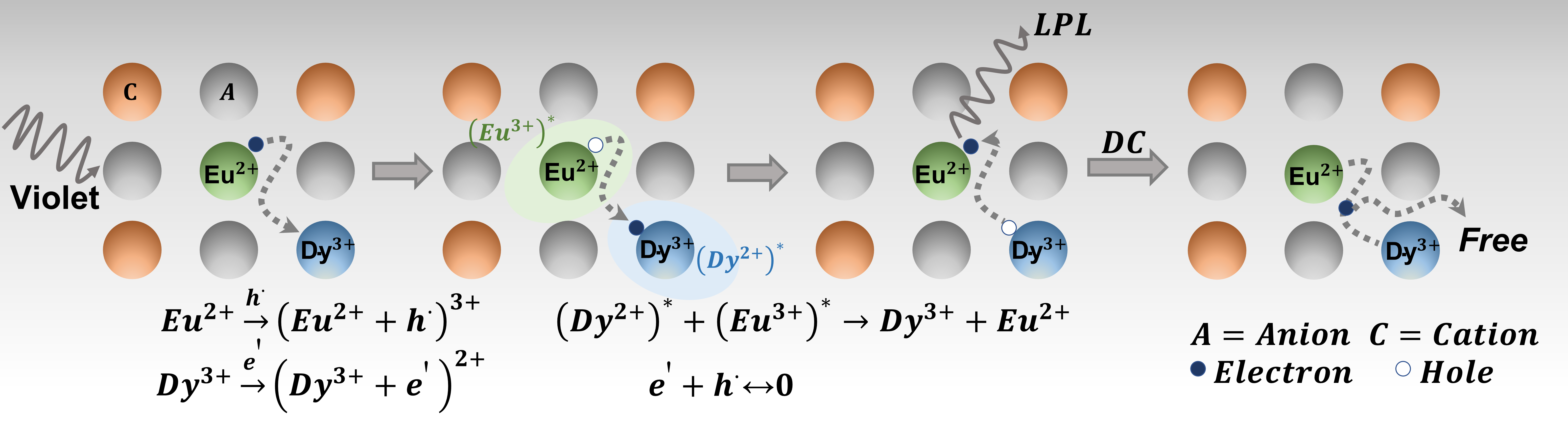
**

**Fig. S14** The proper mechanism of lanthanide ions valence changes during PersL under a low DC electric field at the atomic level.

**Note:** Fig. S14 describes a possible mechanism at the atomic level for four sections: ⅰ) Under UV excitation, the 4f electrons of Eu^2+^ are excited to the 5d orbitals, and it can also be captured by adjacent Dy^3+^. ⅱ) On account of lanthanide ions' electron orbital approximations and the electric neutrality of the lattice, the short-range bound state $\left( {Dy}^{3+}+e^{ꞌ} \right)^{2+}$ principally can form. ⅲ) After stopping the excitation, electrons are released from the $\left( {Dy}^{3+}+e^{ꞌ} \right)^{2+}$ to the 5d orbitals of Eu^2+^ and produces the afterglow. Meanwhile, the bound state of lanthanide ions completes the possible changes of $\left( {Dy}^{2+} \right)^{*}+ \left( {Eu}^{3+} \right)^{*} \to{Dy}^{3+}+ {Eu}^{2+}$ (* refers to the created metastable states). ⅳ) The released carriers can attract to form excitons, which can migrate easily between host lattices^19^ and susceptibly driven by an external electric field, followed by a non-radiative process.

**
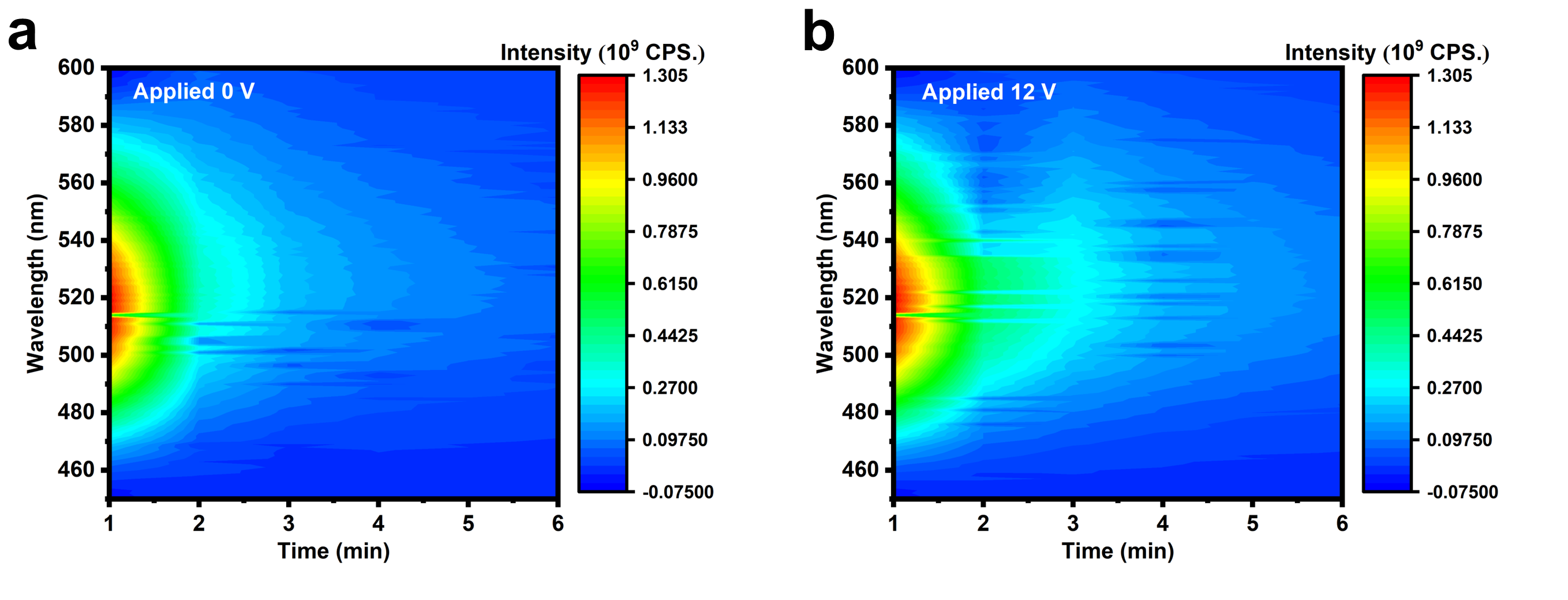
**

**Fig. S15** Voltage-dependent PersL spectra of SAO: Eu^2+^, Dy^3+^ sample depicted in a 3D surface: **a** applied 0 V, **b** applied 12 V.


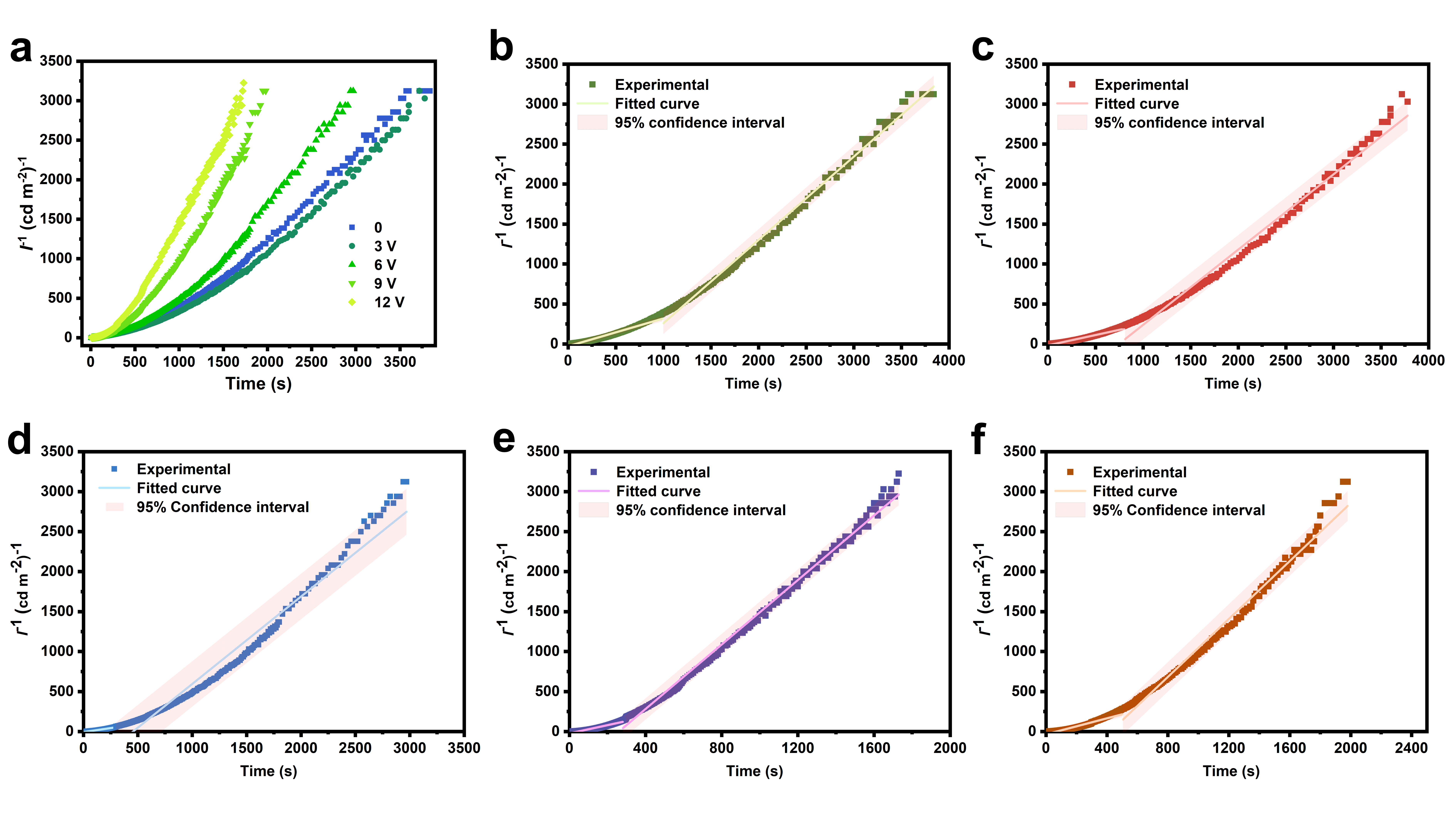


**Fig. S16** The inverse of the afterglow intensity (*I*^-1^) as a function of time when different voltage is applied to SAO:Eu^2+^,Dy^3+^: **a** merge, **b** 0 V, **c** 3 V, **d** 6 V, **e** 9 V, **f** 12 V.

**
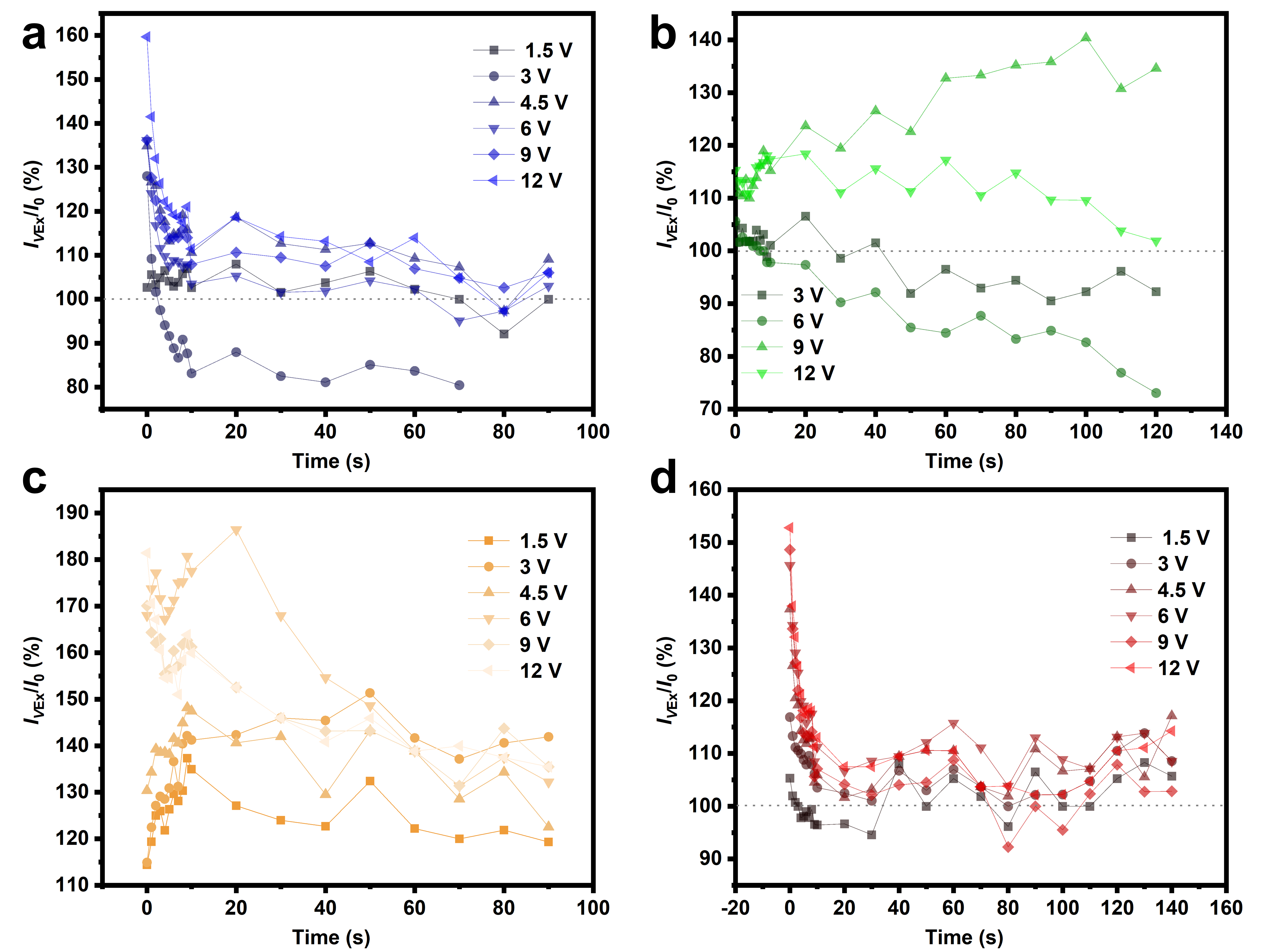
**

**Fig. S17** Comparison of afterglow brightness with or without voltage stimulation: **a** CaAl_2_O_4_:Eu^2+^,Nd^3+^, **b** Ca_6_BaP_4_O_17_:Eu^2+^,Ho^3+^, **c** ZnS:Mn^2+^, **d** Y_2_O_2_S:Eu^3+^.

**
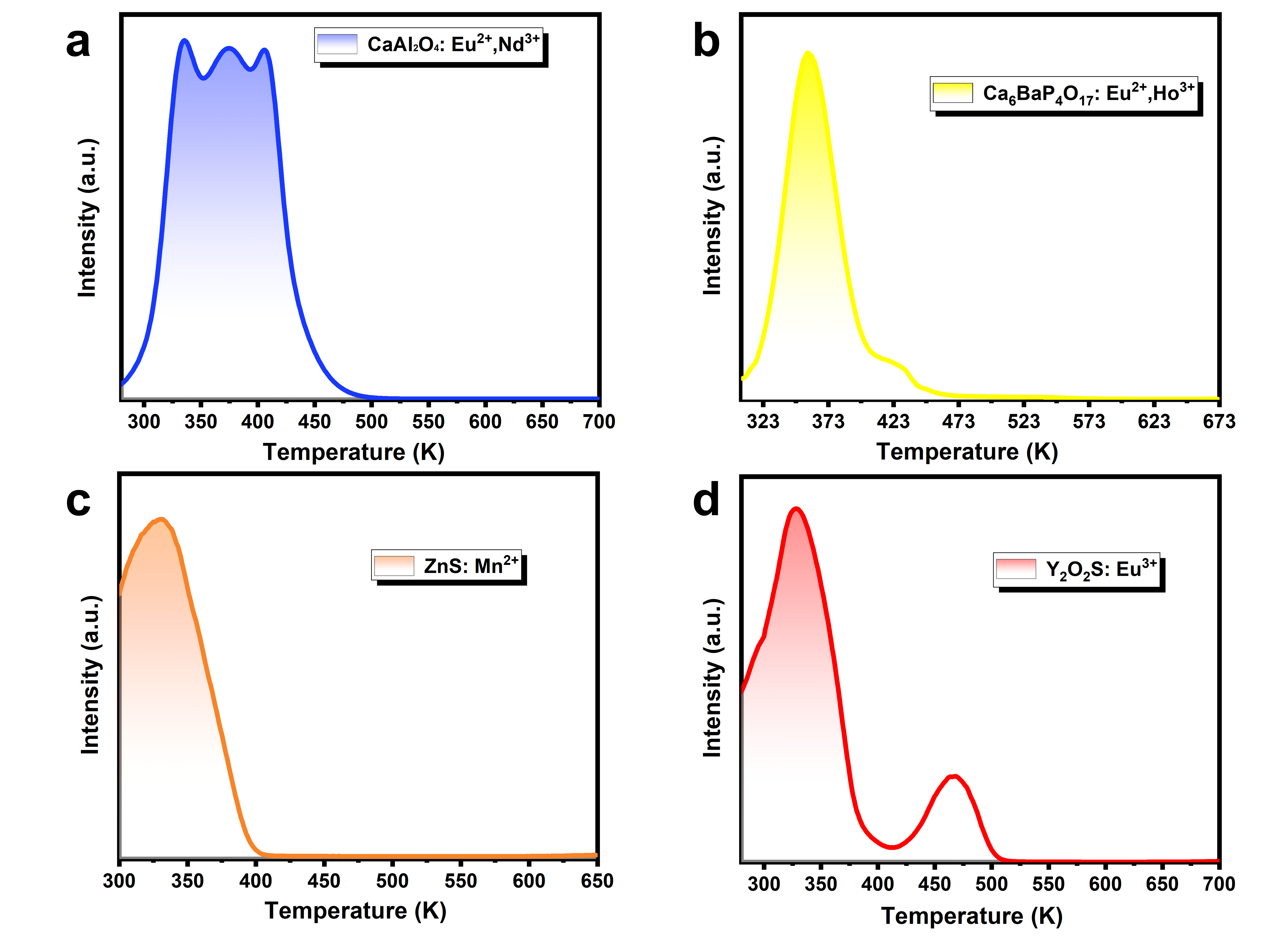
**

**Fig. S18** The related TL spectra: **a** CaAl_2_O_4_:Eu^2+^,Nd^3+^, **b** Ca_6_BaP_4_O_17_:Eu^2+^,Ho^3+^, **c** ZnS:Mn^2+^, **d** Y_2_O_2_S:Eu^3+^.

**Supplementary Tables**

**Table S1.** Crystallographic parameters and Rietveld refinement results of SrAl_2_O_4_:Eu^2+^,Dy^3+^

| Space Group | *P*2_1_ | |
| --- | --- | --- |
| Cell Parameters | a (Å) | 8.415 |
|  | b (Å) | 8.791 |
|  | c (Å) | 5.141 |
|  | α (°) | 90.000 |
|  | β (°) | 93.403 |
|  | γ (°) | 90.000 |
|  | V (Å^3^) | 379.641 |
| Reliability Factors | R_wp_ | 0.1068 |
|  | R_p_ | 0.0801 |
|  | R_B_ | 0.0676 |
|  | χ^2^ | 1.259 |

**Table S2.** Refined atomic coordinates of SrAl_2_O_4_:Eu^2+^,Dy^3+^

| Atom | Wyckoff | x/a | y/b | z/c | Occupancy |
| --- | --- | --- | --- | --- | --- |
| Sr1 | 2a | 0.4925(4) | 0.0027(13) | 0.2478(7) | 0.985(0) |
| Sr2 | 2a | 0.0301(4) | 1.0000(15) | 0.2036(7) | 0.985(0) |
| Al1 | 2a | 0.1788(21) | 0.8288(21) | 0.7169(24) | 1.000(0) |
| Al2 | 2a | 0.8031(19) | 0.8455(15) | 0.7375(24) | 1.000(0) |
| Al3 | 2a | 0.7176(19) | 0.6721(19) | 0.2225(31) | 1.000(0) |
| Al4 | 2a | 0.6828(20) | 0.1742(17) | 0.7955(27) | 1.000(0) |
| O1 | 2a | 0.2800(23) | 0.1844(24) | 0.431(5) | 1.000(0) |
| O2 | 2a | 0.7332(28) | 0.3244(30) | 0.576(6) | 1.000(0) |
| O3 | 2a | 0.3126(15) | 0.494(4) | 0.3609(29) | 1.000(0) |
| O4 | 2a | 0.2812(21) | 0.976(5) | 0.8827(34) | 1.000(0) |
| O5 | 2a | 0.1740(21) | 0.2837(20) | 0.953(4) | 1.000(0) |
| O6 | 2a | 0.1891(28) | 0.6652(26) | 0.894(4) | 1.000(0) |
| O7 | 2a | 0.486(4) | 0.2166(31) | 0.912(5) | 1.000(0) |
| O8 | 2a | 1.0002(25) | 0.8749(22) | 0.6468(35) | 1.000(0) |
| Eu1 | 2a | 0.4925(4) | 0.0027(13) | 0.2478(7) | 0.005(0) |
| Eu2 | 2a | 0.0301(4) | 1.0000(15) | 0.2036(7) | 0.005(0) |
| Dy1 | 2a | 0.4925(4) | 0.0027(13) | 0.2478(7) | 0.01(0) |
| Dy2 | 2a | 0.0301(4) | 1.0000(15) | 0.2036(7) | 0.01(0) |

**Table S3**. Afterglow brightness at different durations

| No. | Chemical Formula | Afterglow brightness (cd m^-2^) | | | | | |
| --- | --- | --- | --- | --- | --- | --- | --- |
|  |  | 0 s | 100 s | 200 s | 300 s | 400 s | 500 s |
| 1 | SrAl_2_O_4_:Eu^2+^ | 0.581 | 0.010 | 0.004 | 0.002 | 0.002 | 0.001 |
| 2 | SrAl_2_O_4_:Eu^2+^,Dy^3+^ | 5.833 | 0.063 | 0.030 | 0.019 | 0.014 | 0.011 |
| 3 | SrAl_2_O_4_:Eu^2+^,Ho^3+^ | 0.257 | 0.022 | 0.0088 | 0.005 | 0.004 | 0.003 |
| 4 | SrAl_2_O_4_:Eu^2+^,Er^3+^ | 0.632 | 0.010 | 0.0046 | 0.003 | 0.002 | 0.002 |
| 5 | SrAl_2_O_4_:Eu^2+^,Tm^3+^ | 0.714 | 0.005 | 0.0022 | 0.004 | 0.001 | 0.001 |
| 6 | SrAl_2_O_4_:Eu^2+^,Yb^3+^ | 0.329 | 0.007 | 0.0032 | 0.002 | 0.002 | 0.001 |
| 7 | SrAl_2_O_4_:Eu^2+^,Sm^3+^ | 0.349 | 0.005 | 0.0020 | 0.001 | 0.001 | 0.001 |
| 8 | SrAl_2_O_4_:Eu^2+^,Pr^3+^ | 0.528 | 0.008 | 0.0035 | 0.002 | 0.001 | 0.001 |
| 9 | SrAl_2_O_4_:Eu^2+^,Tb^3+^ | 0.328 | 0.007 | 0.003 | 0.002 | 0.001 | 0.001 |
| 10 | SrAl_2_O_4_:Eu^2+^,Nd^3+^ | 1.669 | 0.024 | 0.010 | 0.006 | 0.005 | 0.004 |

**Table S4**. Trap depths of lanthanide ions doped SAO samples through TL peak position method.

| Doping centers | Trap depths (eV) | Doping centers | Trap depths (eV) |
| --- | --- | --- | --- |
| Eu^2+^, La^3+^ | 0.70 | La^3+^ | 0.64 |
| Eu^2+^, Ce^3+^ | 0.69 | Ce^3+^ | 0.67 |
| Eu^2+^, Pr^3+^ | 0.65 | Pr^3+^ | 0.67 |
| Eu^2+^, Nd^3+^ | 0.65 | Nd^3+^ | 0.67 |
| Eu^2+^, Sm^3+^ | 0.65 | Sm^3+^ | 0.92 |
| Eu^2+^ | 0.64 | Eu^3+^ | 0.67 |
| Eu^2+^, Gd^3+^ | 0.72 | Gd^3+^ | 0.65, 0.92 |
| Eu^2+^, Tb^3+^ | 0.66 | Tb^3+^ | 0.76, 0.96, 1.02 |
| Eu^2+^, Dy^3+^ | 0.74 | Dy^3+^ | 0.76 |
| Eu^2+^, Ho^3+^ | 0.66 | Ho^3+^ | 0.67, 0.76 |
| Eu^2+^, Er^3+^ | 0.66, 0.80 | Er^3+^ | 0.66 |
| Eu^2+^, Tm^3+^ | 0.65, 0.97 | Tm^3+^ | 0.87 |
| Eu^2+^, Yb^3+^ | 0.65, 0.83 | Yb^3+^ | 0.69 |
| Eu^2+^, Lu^3+^ | 0.69 | Lu^3+^ | 0.67, 1.04 |

**Table S5.** Linear fitting (*I*^-1^=*a*+*bt*) parameters of the afterglow brightness reciprocal (*I*^-1^) to time (*t*)

| Applied Voltage/V | Fitting Range | *a* | *b* | *R*^2^ |
| --- | --- | --- | --- | --- |
| 0 | 10-1000 s | -27.346 | 0.347 | 0.959 |
|  | 1000-4000 s | -782.602 | 1.042 | 0.996 |
| 3 | 10-800 s | -16.452 | 0.259 | 0.962 |
|  | 800-3900 s | -701.673 | 0.941 | 0.986 |
| 6 | 10-600 s | -5.733 | 0.199 | 0.974 |
|  | 600-3000 s | -478.706 | 1.093 | 0.967 |
| 9 | 10-500 s | -14.832 | 0.354 | 0.978 |
|  | 500-1980 s | -765.287 | 1.811 | 0.985 |
| 12 | 10-280 s | -22.320 | 0.516 | 0.976 |
|  | 280-1730 s | -523.473 | 2.016 | 0.994 |

**Table S6**. Test conditions of thermoluminescence (TL) glow curves

| Conditions | TL glow curves |
| --- | --- |
| Pre-treatment | Keep at 673 K for 3 min in the dark |
| Mass of samples | 0.005 g |
| Testing temperature | From room temperature to 600 K |
| Heating Rate | 1 K s^-1^ |
| Irradiation wavelength | 365 nm |
| Power of light | 24 W |
| Pre-irradiation times | 10 s |
| Delay times | 10 s |

**Table S7**. Test conditions of afterglow duration curves

| Conditions | Powder Samples  (Fig. S4, Fig. S7) | Devices  (Fig. 3 and Fig. 6) |
| --- | --- | --- |
| Pre-treatment | Keep at 673 K for 3 min in the dark | |
| Mass of samples | Filling the sample tank(~2 g) | ~0.5 g |
| Testing temperature | Room temperature | Room temperature |
| Irradiation wavelength | 365 nm | 365 nm |
| Power of light | 24 W | 24 W |
| Pre-irradiation times | 10 s | 10 s |
| Delay times | 0 s | 0 s |

**Reference**

1 Guo, B. *et al.* Revealing The Degradation Mechanism of (Sr,Ca)AlSiN_3_:Eu^2+^ Phosphor Aged Under Thermal‐Moisture‐Sulfur Conditions: A Combined Experimental and Ab Initio Study. *Laser & Photonics Reviews* **18**, doi:10.1002/lpor.202300838 (2024).

2 Zeng, W., Wang, Y., Han, S., Chen, W. & Li, G. Investigation on long-persistent luminescence of Ca_2_BO_3_Cl:Eu^2+^,Ln^3+^ (Ln=Nd, Dy, Er). *Optical Materials* **36**, 1819-1821, doi:10.1016/j.optmat.2014.04.030 (2014).

3 Ju, G., Hu, Y., Chen, L., Wang, X. & Mu, Z. Persistent luminescence in CaAl2Si2O8:Eu2+,R3+ (R=Pr, Nd, Dy, Ho and Er). *Journal of Luminescence* **146**, 102-108, doi:10.1016/j.jlumin.2013.09.037 (2014).

4 Guo, H. *et al.* Cyan emissive super-persistent luminescence and thermoluminescence in BaZrSi_3_O_9_:Eu^2+^,Pr^3+^ phosphors. *Journal of Materials Chemistry C* **5**, 2844-2851, doi:10.1039/c7tc00133a (2017).

5 Li, Y., Gecevicius, M. & Qiu, J. Long persistent phosphors--from fundamentals to applications. *Chem Soc Rev* **45**, 2090-2136, doi:10.1039/c5cs00582e (2016).

6 Jia, D., Wang, X.-j., Jia, W. & Yen, W. M. Temperature-dependent photoconductivity of Ce^3+^-doped SrAl_2_O_4_. *Journal of Luminescence* **119-120**, 55-58, doi:10.1016/j.jlumin.2005.12.011 (2006).

7 Han, S., Wang, Y., Zeng, W. & Chen, W. An outlook of rare-earth activated persistent luminescence mechanisms. *Journal of Rare Earths* **34**, 245-250, doi:10.1016/s1002-0721(16)60021-2 (2016).

8 Vitola, V., Millers, D., Bite, I., Smits, K. & Spustaka, A. Recent progress in understanding the persistent luminescence in SrAl_2_O_4_: Eu,Dy. *Materials Science and Technology* **35**, 1661-1677, doi:10.1080/02670836.2019.1649802 (2019).

9 Nazarov, M., Brik, M. G., Spassky, D. & Tsukerblat, B. Crystal field splitting of 5d states and luminescence mechanism in SrAl_2_O_4_: Eu^2+^ phosphor. *Journal of Luminescence* **182**, 79-86, doi:10.1016/j.jlumin.2016.10.015 (2017).

10 Arellano-Tánori, O. *et al.* Persistent luminescence dosimetric properties of UV-irradiated SrAl_2_O_4_:Eu^2+^,Dy^3+^ phosphor. *Journal of Luminescence* **128**, 173-184, doi:10.1016/j.jlumin.2007.07.006 (2008).

11 Ma, X. *et al.* Design of efficient color-tunable long persistent luminescence phosphor BaGa_2_O_4_:Pr^3+^ and its performance enhancement via a trap-induced strategy. *Journal of Materials Chemistry C* **10**, 1105-1117, doi:10.1039/d1tc04763a (2022).

12 Petit, R. R., Michels, S. E., Feng, A. & Smet, P. F. Adding memory to pressure-sensitive phosphors. *Light Sci Appl* **8**, 124, doi:10.1038/s41377-019-0235-x (2019).

13 Ye, Q. *et al.* Designing a novel red to near-infrared persistent phosphor CaMgGe_2_O_6_:Mn^2+^,Sm^3+^ based on a vacuum referred binding energy diagram. *Dalton Transactions* **48**, 11052-11062, doi:10.1039/c9dt01384a (2019).

14 Wang, S., Song, Z. & Liu, Q. Recent progress in Ce^3+^/Eu^2+^-activated LEDs and persistent phosphors: focusing on the local structure and the electronic structure. *Journal of Materials Chemistry C* **11**, 48-96, doi:10.1039/d2tc02639b (2022).

15 Wang, Z., Song, Z., Ning, L. & Liu, Q. Sunlight-activated yellow long persistent luminescence from Nb-doped Sr_3_SiO_5_:Eu^2+^ for warm-color mark applications. *Journal of Materials Chemistry C* **8**, 1143-1150, doi:10.1039/c9tc05880j (2020).

16 Zhang, X., Liu, T., Zhao, F., Zhang, N. & Wang, Y. In-situ-formed Cd and Ag_2_S decorated CdS photocatalyst with boosted charge carrier spatial separation for enhancing UV-vis-NIR photocatalytic hydrogen evolution. *Applied Catalysis B-Environmental* **298**, doi:10.1016/j.apcatb.2021.120620 (2021).

17 Griscom, D. L. ELECTRON-SPIN RESONANCE IN GLASSES .2. MAGNETIC-PROPERTIES. *Journal of Non-Crystalline Solids* **40**, 211-272, doi:10.1016/0022-3093(80)90105-2 (1980).

18 Nazarov, M. *et al.* SrAl_2_O_4_:Eu^2+^ (1%) luminescence under UV, VUV and electron beam excitation. *Optical Materials* **75**, 448-452, doi:10.1016/j.optmat.2017.11.001 (2018).

19 Li, L. *et al.* Mechanism of the trivalent lanthanides' persistent luminescence in wide bandgap materials. *Light-Science & Applications* **11**, doi:10.1038/s41377-022-00736-5 (2022).
